# NUP98 rearrangements in AML: molecular mechanisms and clinical implications

**DOI:** 10.3390/onco3030011

**Published:** 2023-07-18

**Authors:** Sagarajit Mohanty

**Affiliations:** 1 Cancer Biology and Genetics, Memorial Sloan-Kettering Cancer Center, New York, NY 10065, USA

**Keywords:** Leukemia, AML, Translocations, Fusion genes, NUP98 fusions, NUP98::NSD1, NUP98::KDM5A, FLT3-ITD

## Abstract

NUP98 fusions constitute a small subgroup of AML patients and remain a high-risk AML subtype. There are approximately thirty types of NUP98 fusion identified in AML patients. These patients show resistance to currently available therapies and poor clinical outcomes. NUP98 fusions with different fusion partners have oncogenic transformation potential. This review describes how the NUP98 gene acquires oncogenic properties after rearrangement with multiple partners. In the mechanistic part, the formation of nuclear bodies and dysregulation of the HoxA/Meis1 pathway are highlighted. This review also discusses mutational signatures among NUP98 fusions and their significance in leukemogenesis. It also discusses the clinical implications of NUP98 fusions and their associated mutations in AML patients. Furthermore, it highlights therapeutic vulnerabilities in these leukemias that can be exploited as therapeutic strategies. Lastly, this review discusses the gaps in our knowledge regarding NUP98 fusions in AML, as well as future research opportunities.

## Simple Summary

NUP98 rearrangements are frequent events in myeloid malignancies, especially in acute myeloid leukemia (AML). AML patients carrying NUP98 fusions show poor response to standard treatments and adverse outcomes. This review focuses on recent progress in understanding the underlying mechanisms of NUP98 fusion-driven leukemias and the development of therapeutics against them.

## Introduction

Acute myeloid leukemia (AML) is a myeloid malignancy characterized by genomic abnormalities and a high number of blast cells in the bone marrow. [1] Like other cancers, fusion genes are also reported in AML. Fusion genes are formed by chromosome aberrations such as translocations, inversions, deletions, and insertions. [2] Fusion genes can lead to oncogenic transformation by activating oncogenes or inactivating tumor suppressors. [3] Whenever a 3′ oncogene is linked to a strong promoter of a 5′ gene, it becomes overexpressed. In *TMPRSS2::ETS* fusions in prostate cancer, expression of ETS family transcription factor is driven by *TMPRSS2* gene promoter. [4] An oncogene can lose its 3′ UTR microRNA binding site through fusion and lead to higher expression of the oncogene. For example, *MYB::NFIB* fusion in adenoid cystic carcinomas (ACC) activates critical MYB targets through the loss of the 3′ UTR regulating microRNA binding site. [5] Similarly, a gene can inactivate its tumor suppressor function in a fusion.

As a result of recent advances in high throughput sequencing technologies, it has been possible to detect cryptic fusion genes that are usually skipped by conventional karyotyping. [6] For example, one third of *KMT2A* fusions in AML are missed by karyotyping and require additional tests like FISH or RT-PCR. These methods fail to identify rare fusion genes. [7] However, the next generation sequencing (NGS) successfully identifies rare fusions in patient samples. [8, 9] The discovery of these fusion genes has improved the diagnosis, prognosis, and treatment of cancer patients.

In addition, a malignancy caused by a fusion gene opens the door to targeted therapies. Since fusion genes are exclusive to neoplastic cells and not expressed in healthy cells, they are excellent drug targets for treatment. For instance, imatinib was discovered against the *BCR-ABL* fusion gene which is expressed in 95 percent of chronic myeloid leukemia (CML) patients. [10] Discovery of fusion genes in AML helps with risk stratification and treatment of AML patients. Approximately 30–40 percent of AML patients carry at least one fusion gene and *NUP98* fusions frequently occur in AML. [11, 12]

## NUP98: a commonly translocated gene in AML

Nucleoporin 98 (NUP98) is a gene located on chromosome 11p15 and encodes a precursor protein that results in NUP98 and NUP96 nucleoporins, which are structural components of the nuclear pore complex (NPC). [13, 14] NPC facilitates the nucleocytoplasmic transport of ions, mRNAs and proteins. Large molecules are transported via nuclear transport receptors that recognize nuclear export signals (NES) or nuclear localization signals (NLS), while smaller molecules can pass easily through them. [15, 16]

NUP98 is located on both sides of the nuclear pore complex and migrates on and off the NPC. [17] One third of nucleoporins have phenylalanine-glycine (FG) repeats, but NUP98 has a unique FG repeat signature of Gly-Leu-Phe-Gly (GLFG) repeats [18, 19] Besides the GLFG repeats, the N terminal part of the NUP98 protein contains a GLE2-binding sequence (GLEBS) motif, and the C terminal part contains an RNA-binding motif. The GLFG repeats interact with Exportin 1 (XPO1) and mediate nuclear protein export. [20] The GLFG repeats also interact with importin-β family proteins for nuclear import. [19] RNA export factor RAE1 (Gle2) binds to the GLEBS motif to mediate the nuclear export of mRNAs. [21, 22] Additionally, NUP98 is involved in the regulation of transcription. NUP98 is a mobile component of NPC and forms nuclear bodies, known as GLFG bodies. [23] The GLFG repeats interact with histone deacetylases (HDACs) and transcriptional co-activators CBP/p300, suggesting involvement of NUP98 in transcription regulation [24] Further, Kalverda showed altered expression of nucleoplasmic NUP98 affects its target gene expression, supporting its involvement in gene regulation. [25]

*NUP98* gene alterations have been implicated in several hematological malignancies including AML, Chronic myeloid leukemia (CML), Juvenile myelomonocytic leukemia (JMML), T-cell acute lymphoblastic leukemia (T-ALL) and Myelodysplastic syndrome (MDS). [26] Notably, *NUP98* fusions are majorly reported in myeloid and T cell malignancies and rarely observed in B cell malignancies. Around 5% of pediatric AML patients exhibit NUP98 rearrangements.[27–30]

## Fusion partners of NUP98 in NUP98 rearranged AML

The *NUP98* gene is rearranged with approximately 30 partners in AML (Table 1). *NUP98::NSD1* and *NUP98::KDM5A* are frequently occurring *NUP98* fusions in AML. [30] The NUP98 fusion proteins retain the N-terminal of NUP98, which contains GLFG repeats and GLEBS.motif. NUP98 fusion proteins lack the C-terminus portion of NUP98 containing the RNA binding motif. The C-terminus of the fusion protein is contributed by the partner gene. [31]

**Figure 2.**
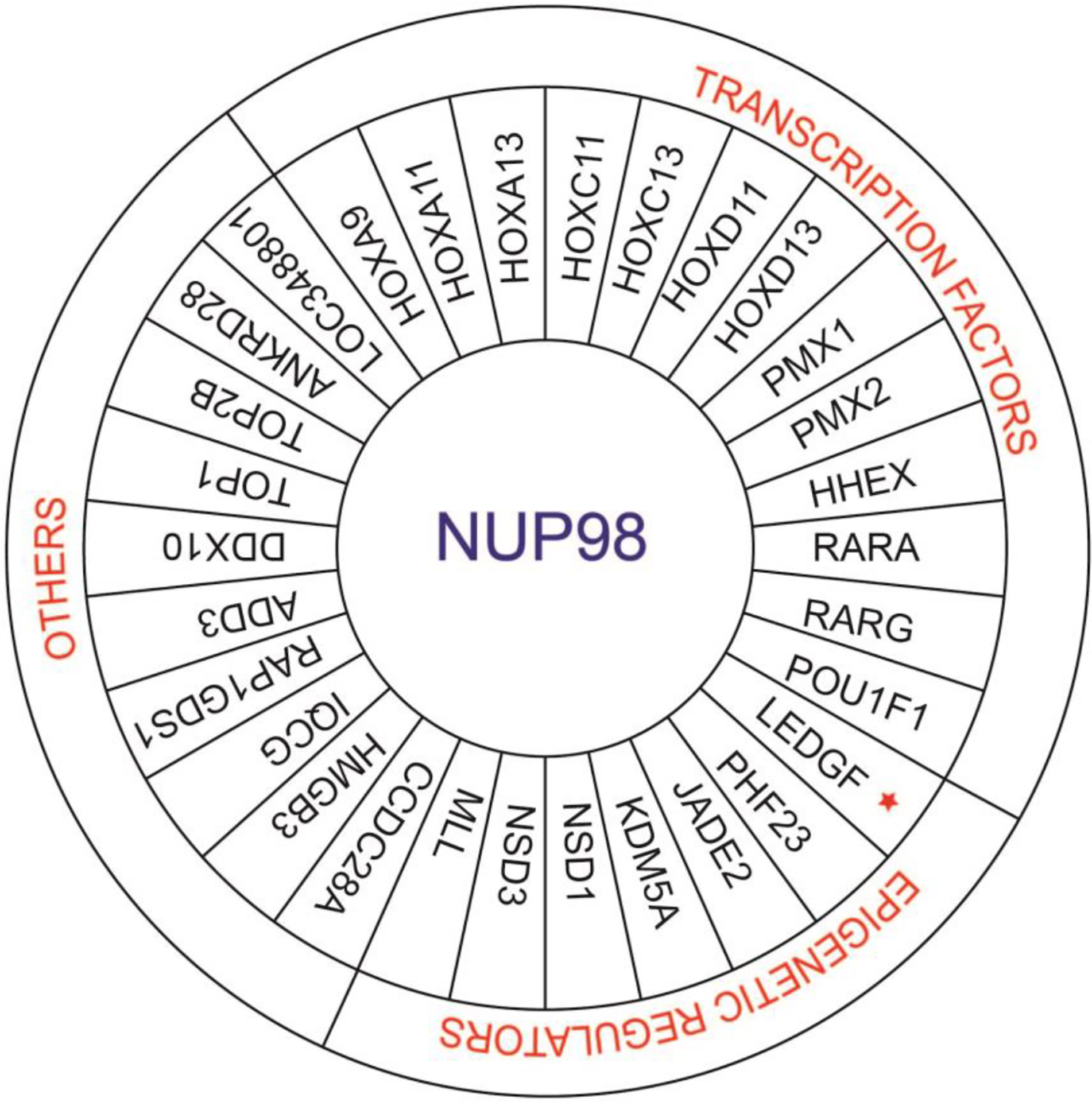
Fusion partners of NUP98 in AML. NUP98 fusion partners can be divided into 3 groups. In the first group, NUP98 has a transcription factor as a fusion partner. In the second group, NUP98 has an epigenetic regulator as a fusion partner. In the second group, the star marked LEDGF is a transcriptional coactivator. The last group includes fusion partners of NUP98 that neither have transcription factor or epigenetic regulation properties.

Overall *NUP98* fusions can be divided into 3 broad parts (Figure 2). The first category includes *NUP98* fusions with transcription factors as partners, which can change the expression of target genes through DNA binding domains. The second category is *NUP98* fusions with epigenetic modifiers that modify chromatin to change target gene expression. The third category of *NUP98* fusions has neither the DNA binding nor chromatin remodeling domain. Transcription factor partners of *NUP98* mostly include homeobox genes, including “class I” HOX genes (*HOXA9*, *HOXA11*, *HOXA13*, *HOXC11*, *HOXC13*, *HOXD11* and *HOXD13*) and “class II” HOX genes *(PMX1*, *PMX2*, *HHEX1* and *POU1F1*) and non-homeobox genes (*RARA* and *RARG*). [26] RARA and RARG are the nuclear receptor (NR) superfamily members. [32, 33] The *LEDGF* (Lens Epithelium-derived Growth Factor) gene encodes p75 and p52, which act as transcriptional coactivators.[34]

NUP98 fusions with epigenetic modifiers typically have plant homeodomain (PHD) domains (PHF23, JADE2, KDM5A, MLL, NSD1, and NSD3) and SET domains (MLL, NSD1 and NSD3). [26, 31] Among the third group, there are a number of partners that have topoisomerase and RNA helicase activities or are involved in signaling activities. (Table1)

AML patients with *NUP98* fusions display different French American and British (FAB) subtypes*. PML::RARA* fusion is usually a characteristic of acute promyelocytic leukemia (APL) or the M3 subtype of AML. [75] However certain AML types with NUP98 translocations like NUP98::JADE2, NUP98::RARA and NUP98::RARG resemble the APL phenotype. [32, 33, 56] The PML-RARA fusion inhibits RARA target genes which blocked differentiation at the promyelocyte stage which leads to APL.[76] This may indicate that the NUP98 rearrangements associated with the APL phenotype prevent the expression of RARA target genes. However, the mechanism of APL transformation and the response to ATRA therapy by these NUP98 fusions is not clearly understood yet. Compared to other NUP98 fusions, the *NUP98::KDM5A* fusion is enriched in the M6/M7 subtype of AML. *NUP98::KDM5A* fusion occurs in about twenty percent of AEL cases. *NUP98-KDM5A* fusion also occurs in approximately ten percent of pediatric acute.megakaryoblastic leukemia (AMKL) cases. [57, 77] Although *NUP98::NSD1* fusion appears in different AML subtypes, it is more frequent in M4/M5 subtypes. [70] *NUP98::HOX* fusions mostly occur in undifferentiated or minimally differentiated AML subtypes (M0, M1 and M2) (Table 1).

## NUP98 fusions represent a poor prognostic and chemoresistant AML subgroup

*NUP98* fusions are associated with adverse clinical outcomes in AML. Patients with *NUP98* rearrangements, predominantly *NUP98::NSD1* fusion, showed poor overall survival (OS) and disease-free survival (DFS) in a pediatric AML cohort. [78] Additionally, more than 70% of *NUP98* fusion positive patients were refractory after the induction therapy. [27] In this line, other studies reported induction failure and chemotherapy resistance in pediatric AML patients carrying *NUP98::NSD1* fusion. [79, 80] Shiba et al demonstrated that *NUP98::NSD1* like patients, with the similar gene expression signature as *NUP98::NSD1*, confer poor overall survival like *NUP98::NSD1* patients. *NUP98::HOXA13*, *DEK::NUP214*, *MLL::MLLT4* were observed in the *NUP98::NSD1* like subgroup. [81] Further, a study by the Children’s Oncology Group (COG) and the European AML study groups demonstrated poor survival and higher relapse risk in *NUP98::KDMA*+ pediatric AML patients. [58] In a study including Acute Erythroid Leukemia (AEL) patients, *NUP98* fusions showed adverse clinical outcomes with estimated OS less than 10 percent. [60] Similarly, *NUP98::KDM5A* fusion showed unfavorable outcomes in pediatric AMKL patients. [77] A report from AIEOP AML group, which includes multiple *NUP98* fusions, observed worse event free survival (EFS) and nearly double relapse rate in *NUP98* fusion positive AML patients compared to AML patients without known mutations. [28] Another study conducted by the French ELAM02 Study Group grouped *NUP98* fusions in an adverse subtype together with mutations in *WT1*, *PHF6* and *RUNX1*. In this study, *KMT2A* rearrangements were classified as intermediate subtypes, whereas *CBF* rearrangements, *NPM1* mutations, and double *CEBPA* mutations were classified as favorable subtypes. [78] Similarly, *NUP98* fusions confer poor prognosis in the adult AML cohort. [70, 82] NUP98 fusions often cooccur with *WT1* and *FLT3-ITD* mutations. Therefore, it is always a question as to how these cooperating mutations affect survival and the response to chemotherapy. Niktoreh et al found that cooccurrence of NUP98 fusion, WT1 and FLT3-ITD mutations or any of these two abnormalities shows significantly low 3 year OS compared to patients with none of these mutations or patients with either one of these mutations. [83] Ostronoff et al reported that the addition of *FLT3* mutations decreases the survival chances of patients with *NUP98::NSD1* AML. [84] An interesting study showed that no *NUP98::NSD1* relapsed AML patients exhibit *FLT3* mutations after chemotherapy, but four out of six exhibit WT1 mutations.[79]

## Mechanism of NUP98 fusion mediated AML

The NUP98 fusions mostly retain the N terminus of NUP98 and C terminus of the partner protein. [85] From the N terminus of NUP98, the GLFG repeats play a crucial role in leukemogenesis through recruiting the transcriptional coactivator complex CBP/p300, [24, 86] but the GLEBS domain is dispensable for leukemogenesis. [87] A mechanistic question is whether NUP98 or its partner gene plays a key role in leukemogenesis. Various studies have shown that NUP98 fusions lose their transformation properties when either partner is deleted. For example, deletion of NUP98 or the SET domain of NSD1 in NUP98-NSD1 fusion prevents myeloid progenitor immortalization. [88] Further overexpression of neither NUP98 nor its partner protein is sufficient for oncogenic transformation. [89, 90] These studies indicate that the fusion protein has unique oncogenic properties in comparison to its associated component.

NUP98 fusions can form distinct nuclear dots, suggesting its involvement in gene regulation. [48, 91, 92] These GLFG nuclear bodies are distinct from Cajal bodies, PML bodies (in APL) and splicing factor speckles. [23] NUP98 fusion proteins can bind CRM1 in a distinct manner from wildtype NUP98, thus preventing transcription factors, such as NFAT and NFKB, from being exported from the nucleus. The NUP98::IQCG, NUP98::HOXA9, and NUP98::DDX10 fusion proteins cause nuclear accumulation of P65, which has the potential to activate the NFKB pathway, which may contribute to the development of leukemia mediated by NUP98 fusion proteins. [92, 93] Recent studies observe that membrane less organelles are formed within the nucleus through liquid–liquid phase separation (LLPS) that facilitate active transcription. [94] NUP98 fusion oncoproteins have intrinsically disordered FG motifs that creates nuclear puncta and promote leukemogenesis through formation of these transcription centers. [95–97]

Different NUP98 fusion proteins regulate *HOX* genes expression to drive leukemogenesis. NUP98 fusions bind near the HOX genes loci and activates its expression through chromatin remodeling. Results from different studies confirm that HoxA/Meis1 pathway is the major mechanism through which NUP98 oncoproteins drive leukemogenesis. The expression of distal *HoxA* cluster genes (*Hoxa7*, *Hoxa9* and *Hoxa10*) and *Meis1* are downregulated as hematopoietic stem and progenitor cells differentiate and overexpression of these promote self-renewal. [86, 98, 99] During differentiation, HOXA genes are silenced through through EZH2, a part of Polycomb Repressive Complex 2 (PRC2), mediated transcriptional repression. [88] Cooperation of *Hoxa9* with *Meis1* causes rapid leukemia induction in mice, indicating a crucial pathway through which leukemogenesis happens. [100] NUP98 fusions activates silenced H*oxA* cluster genes. While histone acetylation, H3K4 and H3K36 methylation around HoxA locus confirms active chromatin, H3K27 marks by polycomb repressor complex silence HoxA genes. [86, 101] NUP98 fusions prevent the H3K27me3 repressive mark and add few activation marks to induce expression of HoxA genes and Meis1. [86] NUP98 fusions acetylate histones through the recruitment of enhancer factors CREB-binding protein (CBP) and p300 by NUP98. [24, 86, 102] NUP98 fusions with chromatin modifier partner changes the chromatin near *HoxA* cluster and *Meis1* locus. Histone H3 Lys 36 (H3K36) methylation on *HoxA* locus by SET domain of NUP98::NSD1 activates distal HoxA gene expression and cause bone marrow (BM) immortalization. [88] NSD2 is translocated in multiple myeloma patients and show its oncogenic activity dependent on dimethylation of histone H3 at lysine 36 (H3K36me2). [103] Other epigenetic modifying partners of NUP98, such as PHF23 or KDM5A, dysregulate Hox genes expression through recognition of H3K4me3/2 marks by plant homeodomain (PHD) finger domain. [102, 104] NSD1 also contains PHD fingers, but it lacks residues that interact with H3K4me3.. [102] Small molecules that inhibit the binding of the PHD domain to H3K4me3 can inhibit leukemogenesis. [104, 105] NUP98 fusions with homeobox partner gene like *NUP98::HOXA9, NUP98::HOXA10, NUP98::HOXD13* cooperate with *Meis1* and cause lethal AML. [106–108] However, overexpression of *Meis1* does not affect the survival of *NUP98::TOP1* induced leukemia mice. [89] It indicates that *NUP98* fusions might have distinct oncogenic potential. However, it is unclear how NUP98::HOX oncogenes collaborate with MEIS1 as compared to NUP98::HOXA9 as only HOXA9 has a MEIS1 binding site. Additionally, the lack of *Hoxa9* does not affect the immortalization properties of *NUP98::HOXA9* fusion oncogene [109] Based on this finding, there may be redundant functions of *Hoxa9* in *NUP98* fusion-mediated leukemogenesis. Other *Hox* genes can also drive leukemogenesis, which could be clarified in future studies. NUP98 fusion carrying patients show upregulation of both HOXA and HOXB cluster genes. [57, 70] But the significance of the upregulation of *HOXB* cluster genes in NUP98 fusion oncoprotein driven AML remains unknown.

In addition to transcriptional regulation, *NUP98* fusions also facilitate aneuploidy.[110] The anaphase promoting complex/cyclosome (APC/C) plays a key role in the transition from metaphase to anaphase during the cell cycle and misregulation of this complex can make the cell susceptible to malignant transformation. [111] APC/C^Cdc20^ ubiquitinates securin, leading to its degradation and activates separase to allow chromosome segregation. Further the spindle checkpoint protects from improper chromosome segregation by inhibiting APC. [112] NUP98 fusion proteins interact with APC/C(Cdc20) mediates aneuploidy through obstructing interaction of the mitotic checkpoint complex to the APC/C(Cdc20) and premature securin degradation. [110, 113].

## Cooperating abnormalities in NUP98 rearranged AML

*NUP98* rearranged AML patients show frequent mutations in signal transduction genes (*FLT3*, *NRAS*, *KRAS* and *KIT*) and *WT1*. [114] Different *NUP98* fusions show different co-occurring mutational signatures. For example, *NUP98::KDM5A* positive AEL cases are often associated with *RB1* deletions. [60] A recent report indicated that del(13q) is a frequent event in *NUP98::KDM5A* AML patients, indicating co-occurrence of *NUP98-KDMA* fusion with RB1 deletion. [30] *FLT3-ITD* mutation is a recurring event in *NUP98::NSD1* positive AML patients. [70, 84, 115] *FLT3-ITD* mutation is also observed in some *NUP98::HOXA9* AML patients. [115, 116] *WT1*, *NRAS*, *KRAS* mutations frequently cooccur with *NUP98::NSD1* and *NUP98::HOXA9*. [78, 117, 118] Patients with NUP98 rearranged AML also have mutations in other genes, such as *ASXL1* and *MYC*.[118, 119]

Multiple subtypes of AML (myeloid, erythroid, and megakaryoblastic) exhibit *NUP98::KDM5A* fusion. *NUP98::KDMA* positive acute megakaryoblastic leukemia (AMKL) cases usually do not show any additional mutations. [57] It is not yet clear whether different types of AML can be attributed to different origins of cells or the presence or absence of specific additional genetic changes. Mice transplanted with hematopoietic stem and progenitor cells (HSPCs) expressing NUP98::KDM5A fusion oncoprotein develop a myeloid leukemia phenotype. [60] This suggests that additional alterations, such as *RB1* deletion, are required for the development of erythroid leukemia, thus explaining why *RB1* deletion occurs concurrently with *NUP98::KDM5A* positive AEL. There is however a need for more studies to clarify how the same fusion can lead to different types of AML.

**Figure 4.**
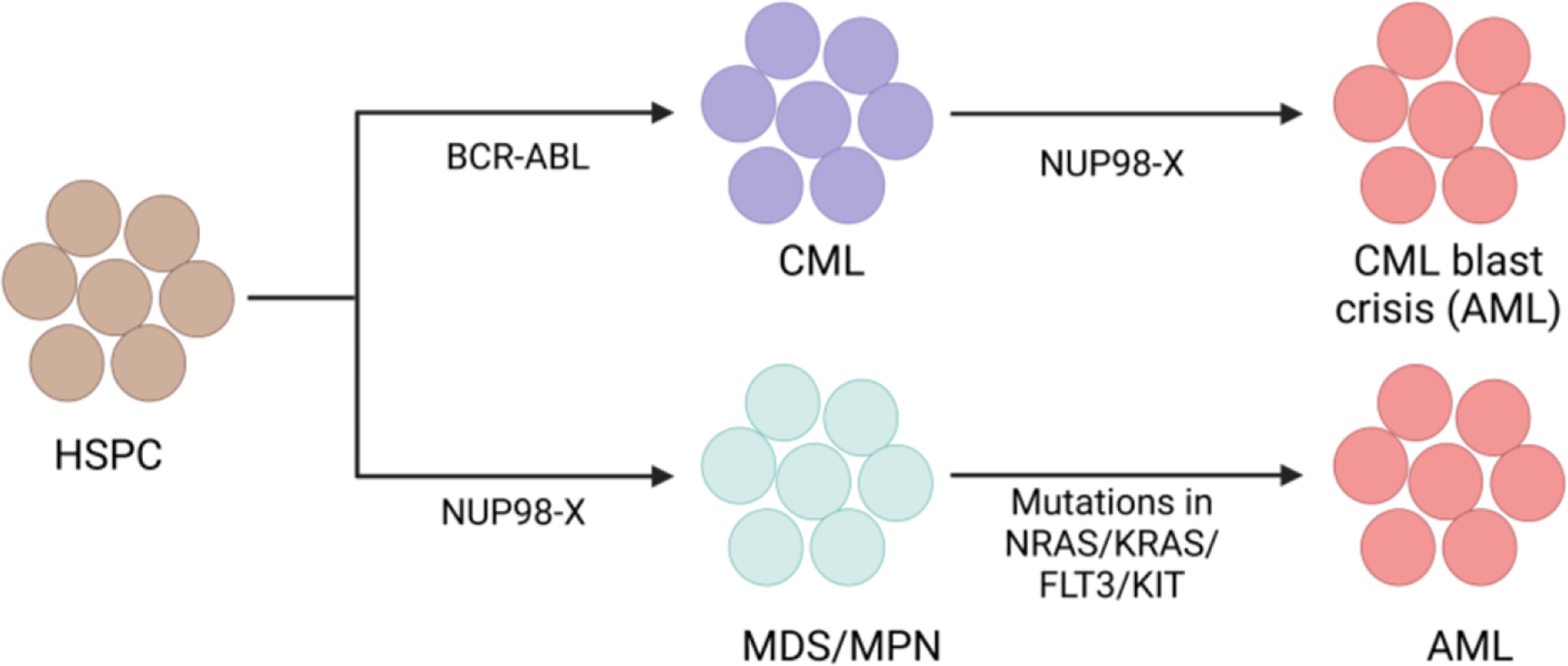
Generalized model of AML development by NUP98 fusions in cooperation with other oncogenic abnormalities. The BCR-ABL oncogene causes a CML like phenotype and undergoes CML blast crisis (AML phenotype) when it acquires a NUP98 fusion oncogene. The NUP98 fusion oncogene shows MDS or MPN phenotype and transforms into AML when it acquires mutations in signaling genes.

One genetic alteration alone does not cause AML; at least two types of genetic alterations must be present for the disease to manifest. While Class- I mutations provide proliferative advantage to cells, Class- II mutations impair differentiation. [120] Class- I mutations include mutations in proliferative genes like *FLT3*, *RAS* or *KIT* but Class- II mutations include different translocations like *MLL* rearrangements, *RUNX1::ETO*, *PML::RARα* fusion. [120, 121] Several studies have demonstrated that *BCR::ABL* positive CML progresses to AML (CML blast crisis) through the acquisition of the *NUP98::HOXA9* fusion gene. [122, 123] Interestingly, another study observed cooperation of *NUP98::HOXA9* with *BCR::ABL* for causing AML with features of CML blast crisis in a murine model. [124] Additionally, NUP*98::HOXA13* and *NUP98::HOXA11* were also reported in CML blast crisis. [37, 124] Another intriguing study observed appearance of *NUP98::DDX10* fusion as a resistance mechanism to Imatinib in CML and caused blast crisis. [125] Fusions involving *NUP98* induce *HOX* gene expression and stem cell renewal, which are considered Class- II mutations*. NUP98* fusions often show mutations in signaling genes like *FLT3*, *NRAS*, *KIT*, indicating the requirement of Class- I mutations for *NUP98* fusion mediated AML development. [114, 117] Different murine model studies have also shown that *NUP98* fusions alone induce long latency myeloid disease, but when collaborates with different proliferative events like *FLT3* or *NRAS* mutations it induces a lethal short latency AML phenotype. [126–130] Retroviral insertional mutagenesis is an excellent tool to identify cooperative events for carcinogenesis. [131] A Retroviral insertional mutagenesis study using *NUP98::HOXD13* fusion showed insertional events near *Meis1*, several signal transduction genes and cell cycle genes. [132] Similarly, another study observed spontaneous mutations in *Nras, Kras* in *NUP98::HOXD13* mice with AML phenotype but these mutations were absent in *NUP98::HOXD13* mice with MDS phenotype [133] Our study demonstrated that addition of NRAS p.G12D mutation further elevates the expression of distal Hoxa genes in NUP98::NSD1 immortalized cells. [128] Overall, the above studies support why signaling mutations are more frequent with *NUP98* rearrangements in AML. (Figure 4) However, additional studies are required to understand the clonal evolution of Future studies can clarify the clonal evoulton pattern of NUP98 fusion mediated AML. Furthr, the studies can explain the mechantic significance of co-occurrence of *NUP98::NSD1* fusion with *WT1* mutation and *NUP98::-KDM5A* fusion with deletion of *RB1*.

## Therapeutic strategies to treat NUP98 fusion positive AML patients

As discussed above, NUP98 fusions show low response to conventional therapies. Therefore, we should explore more targeted therapies for this subset of AML patients. Acute promyelocytic leukemia (APL), a subtype of AML that express *PML::RARα* fusion, is now the most curable AML through targetable degradation of fusion protein using all-trans retinoic acid and (ATRA) and arsenic trioxide (ATO). [134] Therefore, eliminating the NUP98 chimeric protein or its potential downstream target can be developed as a curable treatment option for AML patients carrying NUP98 fusions. There are different ways to target chimeric oncoproteins. In order to eliminate AML, the oncoprotein itself can be targeted directly, or downstream targets of this fusion oncogene or its interactors can be targeted. [135] Several approaches have been proposed for treating NUP98 rearranged AML.

Due to the fact that these fusion genes are expressed only in leukemia cells and not in healthy cells, siRNAs targeting fusion junctions can be designed specifically to target these leukemia cells. Using PDX model, we showed that siRNA lipid nanoparticle formulations can be used as a therapeutic strategy against *NUP98::NSD1* leukemia. [128] This approach is validated against *BCR::ABL, TCF3::PBX1, RUNX1::ETO* fusion oncogenes. [136–138] However, a same fusion protein with different breakpoint can appear as a resistance mechanism to this kind of therapy.

CDK6, which is required for G1 to S phase transition, is upregulated by different NUP98 fusion oncoproteins. NUP98 fusion positive human and mouse samples treated with palbociclib, a CDK4/CDK6 inhibitor, show a reduction in leukemia growth.[67, 139] Different FDA approved CDK 4/6 inhibitors, which are Palbociclib, Ribociclib and Abemaciclib, are used for the treatment of metastatic breast cancer patients. [140]

Previously, it was demonstrated that the menin (MEN1)-MLL interaction is critical for MLL rearranged and NPMc mutant leukemias and small molecules disrupting this interaction are effective against these leukemias. [141, 142]. Disruption of this interaction downregulates leukemic gene expression. Different menin inhibitors (SDNX-5613, JNJ-75276617, BMF-219, DSP 5336 and KO-539) are currently in early-phase clinical trials. [143] A recent study shows that menin-MLL interaction is critical in *NUP98* rearranged AML. VTP50469, an inhibitor of menin-MLL interaction, abrogates leukemogenesis and upregulates differentiation in leukemic cells carrying *NUP98* fusion. Inhibition of menin-MLL interaction downregulates the expression of *Meis1*. [144] Overall, leukemias with KMT2A rearrangements, NUP98 rearrangements, or NPM1 mutations depend upon the HOXA/MEIS1 pathway and are susceptible to menin inhibition. [145] These genotypes account for the majority of patients with AML, indicating that menin inhibitors may have a beneficial effect on a large proportion of AML patients. [145] A recent clinical trial report for revumenib (SNDX-5613), a potent oral menin inhibitor, showed that it was effective in treating relapsed or refractory AML with *KMT2A* rearrangements or *NPM1* mutations. [146] Even though the report indicates promising clinical efficacy, minimal toxicity and well-tolerance for revumenib, clinical resistance could result from mutations in menin that prevent the drug from binding to the target. [147]

The NUP98 fusions interact with XPO1, therefore XPO1 inhibitors may be effective against NUP98 leukemia. [148] Oka et al found that CRM1/XPO1 recruits *Nup98-HoxA9* to *HOX* clusters to drive its expression. [149] Selinexor, an XPO1 inhibitor, has been approved for the treatment of multiple myeloma.[149]

Ren et al showed that the PRC2-KDM5B axis is crucial for NUP98::NSD1 AML. An inhibitor of polycomb repressive complex 2 (PRC2), UNC1999, reduced the leukemic burden in NUP98::NSD1 bearing mice and improved their survival. [150] PRC2 forms silenced chromatin through H3K27me3. The Enhancer of zest homolog 2 (EZH2) is a catalytic subunit of PRC2, so that EZH2 inhibitors can also be tested against NUP98 fusion AML.[151] Tazemetostat, a FDA approved EZH2 inhibitor, is currently used for the treatment of epithelioid sarcoma and follicular lymphoma patients. [152] Another study shows that *NUP98::PHF23* and *NUP98::JARID1A* leukemias are sensitive to disulfiram or a small molecule that inhibits binding of PHF23 plant homeodomain (PHD) motif to H3K4me3 which is essential for *HOXA* and *MEIS1* aberrant expression. [104, 105]

Since NUP98 fusions are frequently associated with other kinase mutations, primarily *FLT3* mutations, kinase inhibitors present an interesting aspect if they are capable of effectively eradicating these leukemic cells. Leukemic cells that express NUP98::NSD1 and FLT3-ITD are effectively inhibited by a FLT3 inhibitor. [129] However, it has been demonstrated that leukemic clones with kinase mutations disappear after chemotherapy. [79] Therefore, kinase inhibitors might alone be insufficient to eliminate major leukemic clones, but it should be used in combination with other inhibitors targeting NUP98 fusions. BCL2 inhibitor navitoclax and SRC/ABL inhibitor dasatinib show synergistic effects against AML cells co-expressing *NUP98::NSD1* and *FLT3-ITD*. [153]

In these AML patients, allogeneic hematopoietic stem cell transplantation (allo-HSCT) is an effective treatment method to prevent relapse. In different studies, relapse was observed despite alloHSCT. [58, 117] According to a study, allo-HSCT in first complete remission (CR1) is more effective than HSCT in second complete remission (CR2) for AML patients with *NUP98::HOXA9* fusion. [154] These studies indicate that allo-HSCT is partial effective for NUP98 rearranged AML patients.

Future research may lead to the development of other treatment options for NUP98-rearranged AML. Since fusion oncoproteins are exclusively expressed on cancer cells, novel ways to target fusion oncoprotein can be explored. The proteolysis-targeting chimeras (PROTACs) technology can be used to target and degrade the fusion oncoprotein and can be developed as a potential treatment approach for NUP98 fusion positive AML patients. [155]. As an ideal source of neoantigens, fusion genes can be exploited for the development of immunotherapy, such as the development of adoptive cell therapy, vaccines, and check point blockade therapy. [156, 157] The benefit of checkpoint blockade therapy for patients with a *NUP98* rearrangement remains undetermined. The identification of unique cell surface proteins in NUP98 fusion subtype AML, which are not expressed in healthy counterparts, may be used for developing chimeric antigen receptor (CAR) T cell therapy. A recent study demonstrated that CD123 is highly expressed in NUP98::NSD1 positive AML patients that can be exploited as an therapeutic target. [158]

## Concluding remarks

Several new fusion partners of *NUP98* have been identified in AML patients in recent years. Although many mechanisms are proposed to explain the leukemogenic activity of NUP98 fusion proteins, HOXA/MES1 appears to be the major player. There is remarkable progress in developing disease models to understand the oncogenic function of *NUP98* rearrangements. These models have been instrumental in validating therapeutic targets and assessing drug responses. Further, the development of patient derived xenograft (PDX) models of NUP98 fusions recapitulates the disease well and is an effective method to track patient cells’ response to different drugs. We currently have PDX models for only a few NUP98 rearrangements, but we will need to establish PDX models for all common NUP98 rearrangements in the near future. [28, 159, 160] In different NUP98 fusion mouse models, siRNA-LNP formulations, CDK6 inhibitor, and menin inhibitor show promising antileukemic activity.

Future studies may focus on discovering new therapeutic vulnerabilities of NUP98 fusions and developing immunotherapies for these patients. Further research into the shared and unique mechanisms of leukemogenic transformation among NUP98 fusion oncoproteins is necessary to clarify whether a single agent can be used for the treatment of all AML patients with different NUP98 rearrangements.

## Figures and Tables

**Figure 1.**
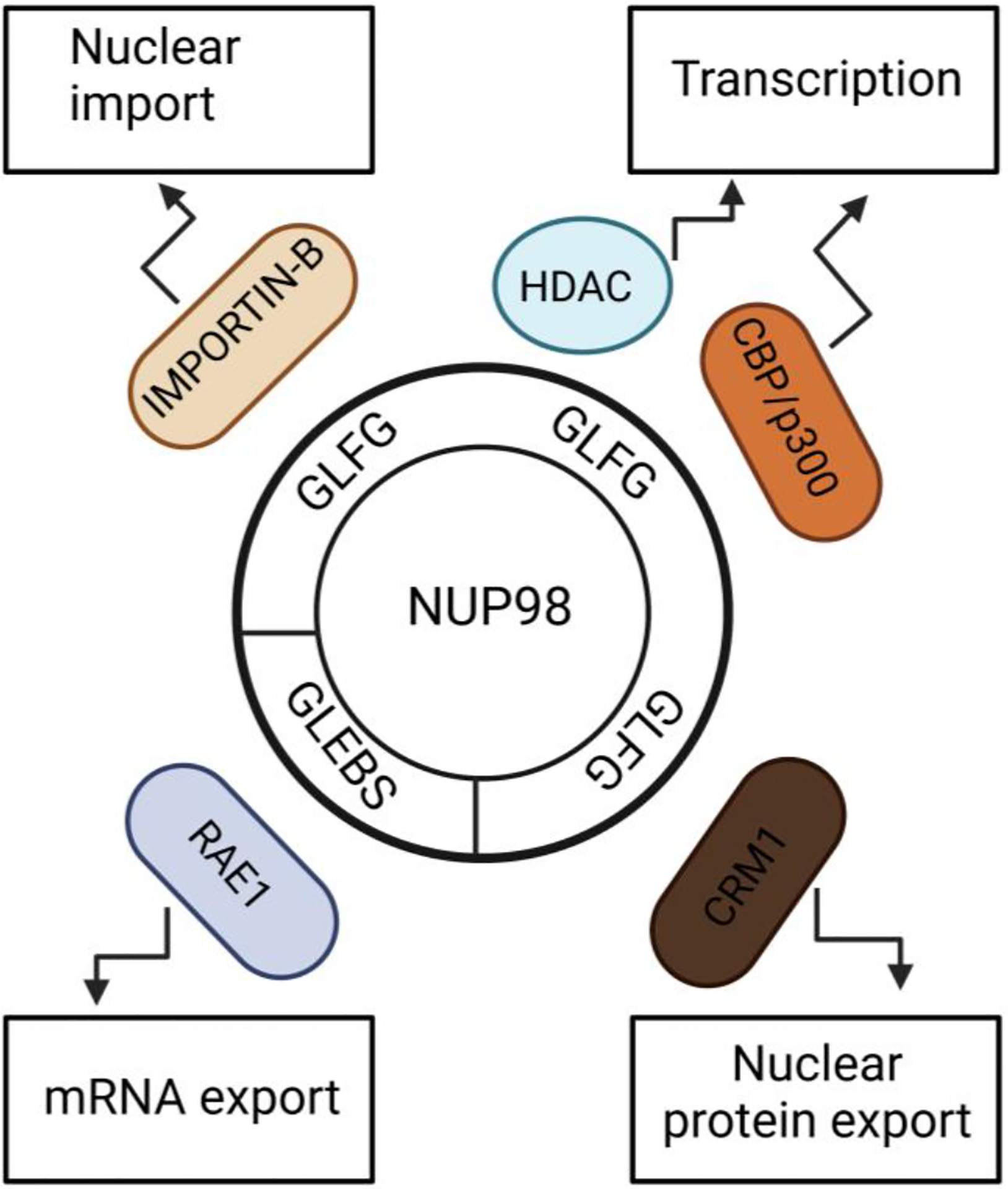
Schematic representation of general functions of important NUP98 protein motifs. GLEBS motif of NUP98 aids nuclear export of mRNAs. GLFG repeats of NUP98 protein are required for multiple functions. It binds to IMPORTIN-B family members for nuclear import, and it binds to CRM1 for nuclear export of proteins. By interacting with HDAC and CBP/p300, it drives gene expression.

**Figure 3.**
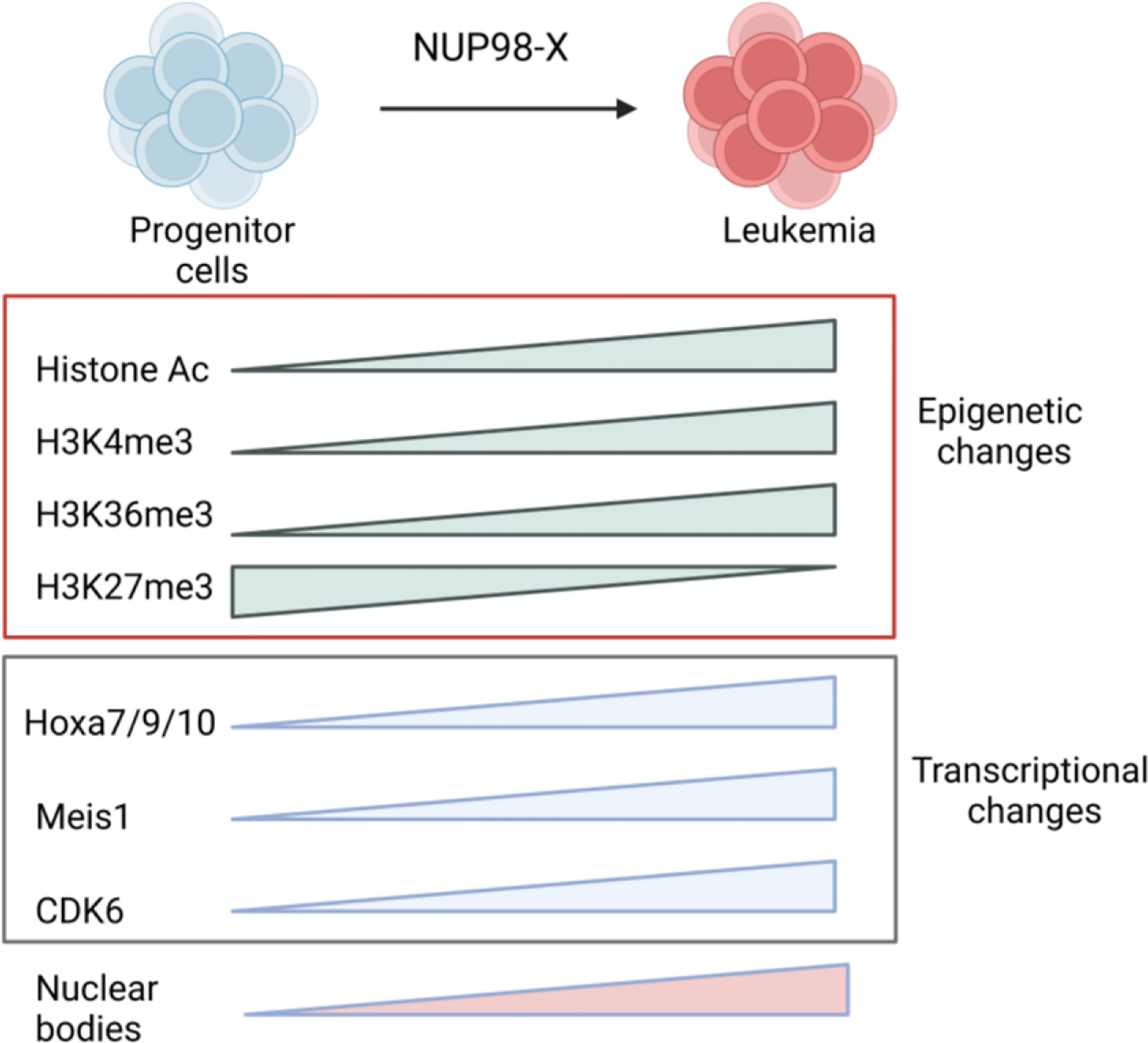
Mechanism of leukemic transformation by NUP98 fusions. Epigenetic and transcriptional changes that occur when Hematopoietic Stem and Progenitor Cells (HSPCs) are transformed using a NUP98 fusion oncogene.

**Figure 5.**
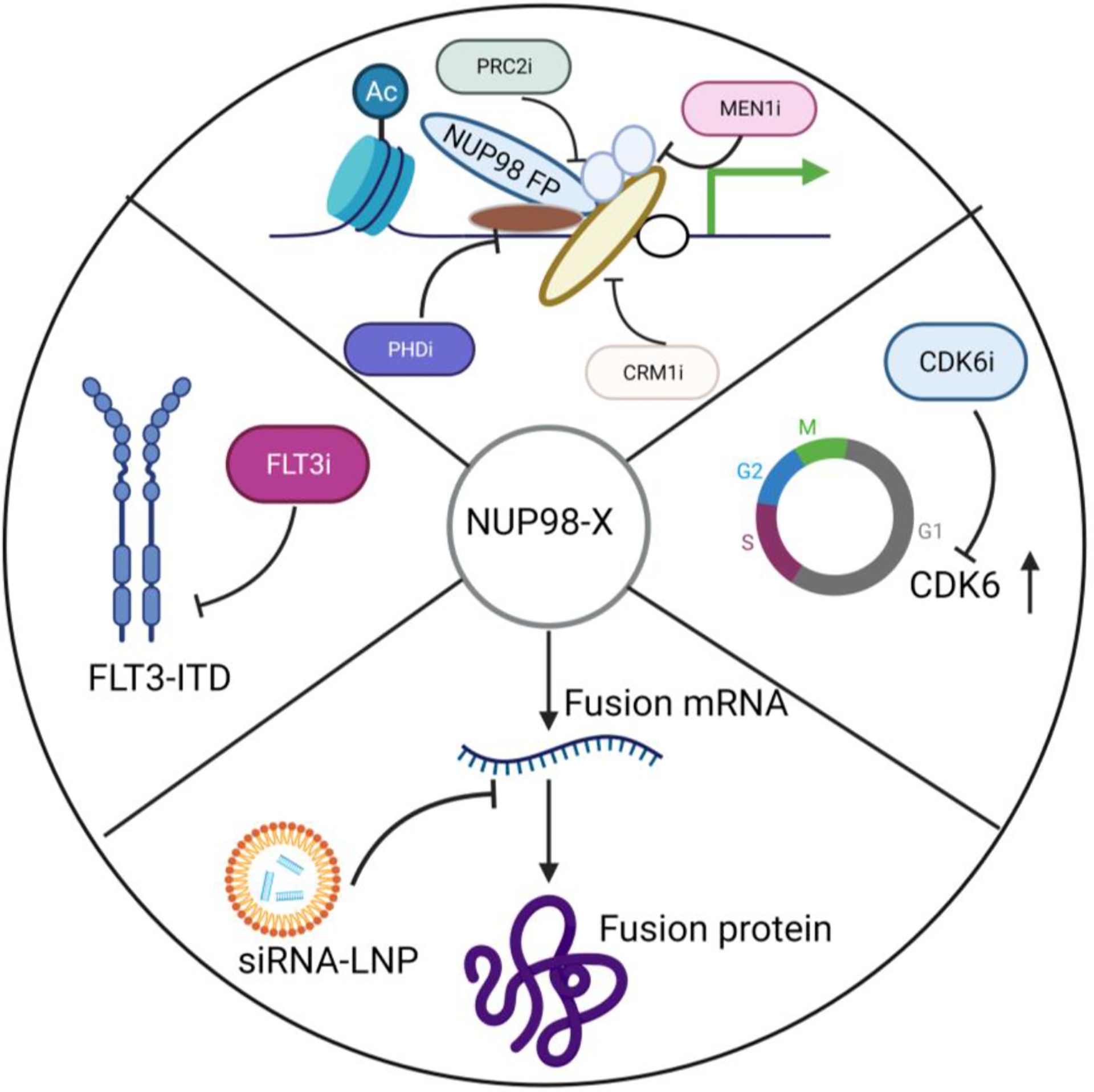
Potential therapeutic options for leukemia with NUP98 rearrangements. NUP98 fusions can be directly targeted using siRNA-LNP formulations. The downstream targets of NUP98 fusion like CDK6 can be inhibited by a small molecule inhibitor. Further CRM1/XPO1, MEN1 or PRC2 inhibitors can be used to eradicate NUP98 fusion driven leukemia. A small molecule inhibitor can be used to inhibit FLT3-ITD mutation that is commonly associated with NUP98 rearrangements.

**Table 1.** Different fusion partners of NUP98 in AML, its functional category and the associated FAB subtypes.

Fusion partner	Functional category	AML subtype	Chromosome rearrangement	REF
HOXA9	Transcription factor	M2, M4	t(7;11)(p15;p15)	[35, 36]
HOXA11	Transcription factor	M2	t(7;11)(p15;p15)	[37]
HOXA13	Transcription factor	M2	t(7;11)(p15;p15)	[38]
HOXC11	Transcription factor	M1,M2, M5	t(11;12)(p15;q13)	[39, 40]
HOXC13	Transcription factor	M2,M4	t(11;12)(p15;q13)	[41, 42]
HOXD11	Transcription factor	M4	t(2;11)(q31;p15)	[43]
HOXD13	Transcription factor	t-AML, M4	t(2;11)(q31;p15)	[44, 45]
*PMX1*	Transcription factor	M2	t(1;11)(q23;p15)	[46]
PMX2	Transcription factor	t-AML	t(9;11)(q34;p15)	[47]
HHEX	Transcription factor	M1, M2	t(10;11)(q23;p15)	[48, 49]
RARA	Transcription factor	M3 or APL	t(11;17)	[32]
RARG	Transcription factor	M3 or APL	t(11;12)(p15;q13)	[33, 50]
POU1F1	Transcription factor	t-AML	t(3;11)(p11;p15)	[51]
LEDGF/PSIP1	Transcription coactivator	M1 and M2 AML	t(9;11)(p22;p15)	[34, 52, 53]
PHF23	Epigenetic modifier	M0,M1,M4 and M5	t(11;17)(p15;p13)	[54, 55]
JADE2/PHF15	Epigenetic modifier	M3 or APL	t(5;11)(q31;p15)	[56]
JARID1A/KDM5A	Epigenetic modifier	M0-M7	t(11;15)(p15;q35)	[57, 58]
NSD1	Epigenetic modifier	M1,M2, M4, M5,M6	t(5;11)(q35;p15.5)	[27, 59–61]
NSD3	Epigenetic modifier	M1	t(8;11)(p11.2;p15)	[62]
MLL/KMT2A	Epigenetic modifier	M1, M2	inv(11)(p15q23)	[63]
C6orf80/CCDC28A	Unknown	M7	t(6;11)(q24.1;p15.5)	[64]
HMGB3	high mobility group (HMG) protein	t-AML	t(X;11)(q28;p15)	[65]
IQCG	Calcium signalling	AML(Unknown)	t(3;11)(q29q13;p15)	[66]
RAP1GDS1	GTPase activity	AML(Unknown)	unknown	[67]
ADD3	Cytoskeletal protein	AML(Unknown)	t(10;11)	[68]
DDX10	RNA helicase	M6	inv(11)(p15q22)	[69, 70]
TOP1	DNA Topoisomerase	M4, M5	t(11;20)(p15;q11)	[27, 71]
TOP2B	DNA Topoisomerase	M5	t(3;11)(p24;p15)	[72]
ANKRD28	Signalling protein	AML(Unknown)	t(3;5;11)(p25;q35;p15)	[73]
LOC348801	Unknown	M2	t(3;11)(q12;p15)	[74]

Undifferentiated acute myeloblastic leukemia (M0), Acute myeloblastic leukemia with minimal maturation (M1), Acute myeloblastic leukemia with maturation (M2), Acute promyelocytic leukemia (APL) (M3), Acute myelomonocytic leukemia (M4), Acute monocytic leukemia (M5), Acute erythroid leukemia (AEL) (M6), Acute megakaryoblastic leukemia (M7), Therapy related acute myeloid leukemia (t-AML).

## References

[1] Khoury JD, Solary E, Abla O, Akkari Y, Alaggio R, Apperley JF, Bejar R, Berti E, Busque L, Chan JKC, Chen W, Chen X, Chng W-J, Choi JK, Colmenero I, Coupland SE, Cross NCP, De Jong D, Elghetany MT, Takahashi E, Emile J-F, Ferry J, Fogelstrand L, Fontenay M, Germing U, Gujral S, Haferlach T, Harrison C, Hodge JC, Hu S, Jansen JH, Kanagal-Shamanna R, Kantarjian HM, Kratz CP, Li X-Q, Lim MS, Loeb K, Loghavi S, Marcogliese A, Meshinchi S, Michaels P, Naresh KN, Natkunam Y, Nejati R, Ott G, Padron E, Patel KP, Patkar N, Picarsic J, Platzbecker U, Roberts I, Schuh A, Sewell W, Siebert R, Tembhare P, Tyner J, Verstovsek S, Wang W, Wood B, Xiao W, Yeung C, Hochhaus A. The 5th edition of the World Health Organization Classification of Haematolymphoid Tumours: Myeloid and Histiocytic/Dendritic Neoplasms. Leukemia. 2022;36(7):1703–19. 35732831 10.1038/s41375-022-01613-1 PMC9252913

[2] Taniue K, Akimitsu N. Fusion Genes and RNAs in Cancer Development. Non-coding RNA. 2021;7(1). 10.3390/ncrna7010010 PMC7931065 33557176

[3] Gao Q, Liang WW, Foltz SM, Mutharasu G, Jayasinghe RG, Cao S, Liao WW, Reynolds SM, Wyczalkowski MA, Yao L, Yu L, Sun SQ, Chen K, Lazar AJ, Fields RC, Wendl MC, Van Tine BA, Vij R, Chen F, Nykter M, Shmulevich I, Ding L. Driver Fusions and Their Implications in the Development and Treatment of Human Cancers. Cell Rep. 2018;23(1):227–38.e3. 29617662 10.1016/j.celrep.2018.03.050 PMC5916809

[4] Tu JJ, Rohan S, Kao J, Kitabayashi N, Mathew S, Chen Y-T. Gene fusions between TMPRSS2 and ETS family genes in prostate cancer: frequency and transcript variant analysis by RT-PCR and FISH on paraffin-embedded tissues. Modern Pathology. 2007;20(9):921–8. 17632455 10.1038/modpathol.3800903

[5] Persson M, Andrén Y, Mark J, Horlings HM, Persson F, Stenman G. Recurrent fusion of MYB and NFIB transcription factor genes in carcinomas of the breast and head and neck. Proc Natl Acad Sci U S A. 2009;106(44):18740–4. 19841262 10.1073/pnas.0909114106 PMC2773970

[6] Annala MJ, Parker BC, Zhang W, Nykter M. Fusion genes and their discovery using high throughput sequencing. Cancer letters. 2013;340(2):192–200. 23376639 10.1016/j.canlet.2013.01.011 PMC3675181

[7] Engvall M, Cahill N, Jonsson B-I, Höglund M, Hallböök H, Cavelier L. Detection of leukemia gene fusions by targeted RNA-sequencing in routine diagnostics. BMC Medical Genomics. 2020;13(1):106. 32727569 10.1186/s12920-020-00739-4 PMC7388219

[8] Heydt C, Wölwer CB, Velazquez Camacho O, Wagener-Ryczek S, Pappesch R, Siemanowski J, Rehker J, Haller F, Agaimy A, Worm K, Herold T, Pfarr N, Weichert W, Kirchner T, Jung A, Kumbrink J, Goering W, Esposito I, Buettner R, Hillmer AM, Merkelbach-Bruse S. Detection of gene fusions using targeted next-generation sequencing: a comparative evaluation. BMC Medical Genomics. 2021;14(1):62. 33639937 10.1186/s12920-021-00909-y PMC7912891

[9] Padella A, Simonetti G, Paciello G, Giotopoulos G, Baldazzi C, Righi S, Ghetti M, Stengel A, Guadagnuolo V, De Tommaso R, Papayannidis C, Robustelli V, Franchini E, Rorà A, Ferrari A, Fontana MC, Bruno S, Ottaviani E, Soverini S, Storlazzi CT, Haferlach C, Sabattini E, Testoni N, Iacobucci I, Huntly BJP, Ficarra E, Martinelli G. Novel and Rare Fusion Transcripts Involving Transcription Factors and Tumor Suppressor Genes in Acute Myeloid Leukemia. Cancers (Basel). 2019;11(12). 10.3390/cancers11121951 PMC6966504 31817495

[10] Druker BJ, Tamura S, Buchdunger E, Ohno S, Segal GM, Fanning S, Zimmermann J, Lydon NB. Effects of a selective inhibitor of the Abl tyrosine kinase on the growth of Bcr-Abl positive cells. Nat Med. 1996;2(5):561–6. 8616716 10.1038/nm0596-561

[11] Stengel A, Shahswar R, Haferlach T, Walter W, Hutter S, Meggendorfer M, Kern W, Haferlach C. Whole transcriptome sequencing detects a large number of novel fusion transcripts in patients with AML and MDS. Blood advances. 2020;4(21):5393–401. 33147338 10.1182/bloodadvances.2020003007 PMC7656918

[12] Ley TJ, Miller C, Ding L, Raphael BJ, Mungall AJ, Robertson A, Hoadley K, Triche TJ Jr., Laird PW, Baty JD, Fulton LL, Fulton R, Heath SE, Kalicki-Veizer J, Kandoth C, Klco JM, Koboldt DC, Kanchi KL, Kulkarni S, Lamprecht TL, Larson DE, Lin L, Lu C, McLellan MD, McMichael JF, Payton J, Schmidt H, Spencer DH, Tomasson MH, Wallis JW, Wartman LD, Watson MA, Welch J, Wendl MC, Ally A, Balasundaram M, Birol I, Butterfield Y, Chiu R, Chu A, Chuah E, Chun HJ, Corbett R, Dhalla N, Guin R, He A, Hirst C, Hirst M, Holt RA, Jones S, Karsan A, Lee D, Li HI, Marra MA, Mayo M, Moore RA, Mungall K, Parker J, Pleasance E, Plettner P, Schein J, Stoll D, Swanson L, Tam A, Thiessen N, Varhol R, Wye N, Zhao Y, Gabriel S, Getz G, Sougnez C, Zou L, Leiserson MD, Vandin F, Wu HT, Applebaum F, Baylin SB, Akbani R, Broom BM, Chen K, Motter TC, Nguyen K, Weinstein JN, Zhang N, Ferguson ML, Adams C, Black A, Bowen J, Gastier-Foster J, Grossman T, Lichtenberg T, Wise L, Davidsen T, Demchok JA, Shaw KR, Sheth M, Sofia HJ, Yang L, Downing JR, Eley G. Genomic and epigenomic landscapes of adult de novo acute myeloid leukemia. N Engl J Med. 2013;368(22):2059–74. 23634996 10.1056/NEJMoa1301689 PMC3767041

[13] Wu X, Kasper LH, Mantcheva RT, Mantchev GT, Springett MJ, van Deursen JM. Disruption of the FG nucleoporin NUP98 causes selective changes in nuclear pore complex stoichiometry and function. Proc Natl Acad Sci U S A. 2001;98(6):3191–6. 11248054 10.1073/pnas.051631598 PMC30629

[14] Fontoura BM, Blobel G, Matunis MJ. A conserved biogenesis pathway for nucleoporins: proteolytic processing of a 186-kilodalton precursor generates Nup98 and the novel nucleoporin, Nup96. The Journal of cell biology. 1999;144(6):1097–112. 10087256 10.1083/jcb.144.6.1097 PMC2150585

[15] Paci G, Caria J, Lemke EA. Cargo transport through the nuclear pore complex at a glance. Journal of Cell Science. 2021;134(2). 10.1242/jcs.247874 33495357

[16] Nofrini V, Di Giacomo D, Mecucci C. Nucleoporin genes in human diseases. European journal of human genetics : EJHG. 2016;24(10):1388–95. 27071718 10.1038/ejhg.2016.25 PMC5027676

[17] Griffis ER, Xu S, Powers MA. Nup98 localizes to both nuclear and cytoplasmic sides of the nuclear pore and binds to two distinct nucleoporin subcomplexes. Molecular biology of the cell. 2003;14(2):600–10. 12589057 10.1091/mbc.E02-09-0582 PMC149995

[18] Chatel G, Desai SH, Mattheyses AL, Powers MA, Fahrenkrog B. Domain topology of nucleoporin Nup98 within the nuclear pore complex. Journal of Structural Biology. 2012;177(1):81–9. 22100335 10.1016/j.jsb.2011.11.004 PMC3418325

[19] Iwamoto M, Asakawa H, Hiraoka Y, Haraguchi T. Nucleoporin Nup98: a gatekeeper in the eukaryotic kingdoms. Genes to cells : devoted to molecular & cellular mechanisms. 2010;15(7):661–9. 20545767 10.1111/j.1365-2443.2010.01415.x

[20] Oka M, Asally M, Yasuda Y, Ogawa Y, Tachibana T, Yoneda Y. The mobile FG nucleoporin Nup98 is a cofactor for Crm1-dependent protein export. Molecular biology of the cell. 2010;21(11):1885–96. 20375145 10.1091/mbc.E09-12-1041 PMC2877646

[21] Pritchard CE, Fornerod M, Kasper LH, van Deursen JM. RAE1 is a shuttling mRNA export factor that binds to a GLEBS-like NUP98 motif at the nuclear pore complex through multiple domains. The Journal of cell biology. 1999;145(2):237–54. 10209021 10.1083/jcb.145.2.237 PMC2133102

[22] Ren Y, Seo HS, Blobel G, Hoelz A. Structural and functional analysis of the interaction between the nucleoporin Nup98 and the mRNA export factor Rae1. Proc Natl Acad Sci U S A. 2010;107(23):10406–11. 20498086 10.1073/pnas.1005389107 PMC2890840

[23] Griffis ER, Altan N, Lippincott-Schwartz J, Powers MA. Nup98 is a mobile nucleoporin with transcription-dependent dynamics. Molecular biology of the cell. 2002;13(4):1282–97. 11950939 10.1091/mbc.01-11-0538 PMC102269

[24] Kasper LH, Brindle PK, Schnabel CA, Pritchard CE, Cleary ML, van Deursen JM. CREB binding protein interacts with nucleoporin-specific FG repeats that activate transcription and mediate NUP98-HOXA9 oncogenicity. Mol Cell Biol. 1999;19(1):764–76. 9858599 10.1128/mcb.19.1.764 PMC83933

[25] Kalverda B, Pickersgill H, Shloma VV, Fornerod M. Nucleoporins directly stimulate expression of developmental and cell-cycle genes inside the nucleoplasm. Cell. 2010;140(3):360–71. 20144760 10.1016/j.cell.2010.01.011

[26] Michmerhuizen NL, Klco JM, Mullighan CG. Mechanistic insights and potential therapeutic approaches for NUP98-rearranged hematologic malignancies. Blood. 2020;136(20):2275–89. 32766874 10.1182/blood.2020007093 PMC7702474

[27] Struski S, Lagarde S, Bories P, Puiseux C, Prade N, Cuccuini W, Pages MP, Bidet A, Gervais C, Lafage-Pochitaloff M, Roche-Lestienne C, Barin C, Penther D, Nadal N, Radford-Weiss I, Collonge-Rame MA, Gaillard B, Mugneret F, Lefebvre C, Bart-Delabesse E, Petit A, Leverger G, Broccardo C, Luquet I, Pasquet M, Delabesse E. NUP98 is rearranged in 3.8% of pediatric AML forming a clinical and molecular homogenous group with a poor prognosis. Leukemia. 2017;31(3):565–72. 27694926 10.1038/leu.2016.267

[28] Bisio V, Zampini M, Tregnago C, Manara E, Salsi V, Di Meglio A, Masetti R, Togni M, Di Giacomo D, Minuzzo S, Leszl A, Zappavigna V, Rondelli R, Mecucci C, Pession A, Locatelli F, Basso G, Pigazzi M. NUP98-fusion transcripts characterize different biological entities within acute myeloid leukemia: a report from the AIEOP-AML group. Leukemia. 2017;31(4):974–7. 27890935 10.1038/leu.2016.361

[29] Bertrums EJM, Smith JL, Ries RE, Alonzo TA, Ostronoff F, Kaspers GJL, Hasle H, Zwaan CM, Hirsch BA, Raimondi SC, Cooper TM, Aplenc R, Gamis AS, Kolb EA, Goemans BF, Meshinchi S. The Molecular Characteristics and Clinical Relevance of NUP98-Other Translocations in Pediatric Acute Myeloid Leukemia. Blood. 2020;136:36–7.32430502

[30] Bertrums EJM, Smith JL, Harmon L, Ries RE, Wang YJ, Alonzo TA, Menssen AJ, Chisholm KM, Leonti AR, Tarlock K, Ostronoff F, Pogosova-Agadjanyan EL, Kaspers GJL, Hasle H, Dworzak M, Walter C, Muhlegger N, Morerio C, Pardo L, Hirsch B, Raimondi S, Cooper TM, Aplenc R, Gamis AS, Kolb EA, Farrar JE, Stirewalt D, Ma X, Shaw TI, Furlan SN, Brodersen LE, Loken MR, Van den Heuvel-Eibrink MM, Zwaan CM, Triche TJ, Goemans BF, Meshinchi S. Comprehensive molecular and clinical characterization of NUP98 fusions in pediatric acute myeloid leukemia. Haematologica. 2023. 10.3324/haematol.2022.281653 PMC10388277 36815378

[31] Gough SM, Slape CI, Aplan PD. NUP98 gene fusions and hematopoietic malignancies: common themes and new biologic insights. Blood. 2011;118(24):6247–57. 21948299 10.1182/blood-2011-07-328880 PMC3236115

[32] Zhu HH, Yang MC, Wang F, Lou YJ, Jin J, Li K, Zhang SZ. Identification of a novel NUP98-RARA fusion transcript as the 14th variant of acute promyelocytic leukemia. American journal of hematology. 2020;95(7):E184–e6. 32242976 10.1002/ajh.25807

[33] Such E, Cervera J, Valencia A, Barragán E, Ibañez M, Luna I, Fuster Ó, Perez-Sirvent ML, Senent L, Sempere A, Martinez J, Martín-Aragonés G, Sanz MA. A novel NUP98/RARG gene fusion in acute myeloid leukemia resembling acute promyelocytic leukemia. Blood. 2011;117(1):242–5. 20935257 10.1182/blood-2010-06-291658

[34] Gallego Hernanz MP, Torregrosa Diaz JM, Sorel N, Bobin A, Dindinaud E, Bouyer S, Desmier D, Brizard F, Leleu X, Maillard N, Chomel J-C. Long-term molecular remission in a patient with acute myeloid leukemia harboring a new NUP98-LEDGF rearrangement. 2019;8(4):1765–70. 10.1002/cam4.2051 PMC6488106 30848074

[35] Huang SY, Tang JL, Liang YJ, Wang CH, Chen YC, Tien HF. Clinical, haematological and molecular studies in patients with chromosome translocation t(7;11): a study of four Chinese patients in Taiwan. British journal of haematology. 1997;96(4):682–7. 9074407 10.1046/j.1365-2141.1997.d01-2100.x

[36] Lahortiga I, Belloni E, Vázquez I, Agirre X, Larrayoz MJ, Vizmanos JL, Valgañón M, Zudaire I, Sáez B, Mateos MC, Di Fiore PP, Calasanz MJ, Odero MD. NUP98 is fused to HOXA9 in a variant complex t(7;11;13;17) in a patient with AML-M2. Cancer genetics and cytogenetics. 2005;157(2):151–6. 15721637 10.1016/j.cancergencyto.2004.08.001

[37] Suzuki A, Ito Y, Sashida G, Honda S, Katagiri T, Fujino T, Nakamura T, Ohyashiki K. t(7;11)(p15;p15) Chronic myeloid leukaemia developed into blastic transformation showing a novel NUP98/HOXA11 fusion. British journal of haematology. 2002;116(1):170–2. 11841413 10.1046/j.1365-2141.2002.03246.x

[38] Taketani T, Taki T, Ono R, Kobayashi Y, Ida K, Hayashi Y. The chromosome translocation t(7;11)(p15;p15) in acute myeloid leukemia results in fusion of the NUP98 gene with a HOXA cluster gene, HOXA13, but not HOXA9. Genes Chromosomes Cancer. 2002;34(4):437–43. 12112533 10.1002/gcc.10077

[39] Taketani T, Taki T, Shibuya N, Kikuchi A, Hanada R, Hayashi Y. Novel NUP98-HOXC11 Fusion Gene Resulted from a Chromosomal Break within Exon 1 of HOXC11 in Acute Myeloid Leukemia with t(11;12)(p15;q13)1. Cancer Research. 2002;62(16):4571–4. 12183408

[40] Gu BW, Wang Q, Wang JM, Xue YQ, Fang J, Wong KF, Chen B, Shi ZZ, Shi JY, Bai XT, Wu DH, Chen Z, Chen SJ. Major form of NUP98/HOXC11 fusion in adult AML with t(11;12)(p15;q13) translocation exhibits aberrant trans-regulatory activity. Leukemia. 2003;17(9):1858–64. 12970787 10.1038/sj.leu.2403036

[41] Panagopoulos I, Isaksson M, Billström R, Strömbeck B, Mitelman F, Johansson B. Fusion of the NUP98 gene and the homeobox gene HOXC13 in acute myeloid leukemia with t(11;12)(p15;q13). Genes Chromosomes Cancer. 2003;36(1):107–12. 12461755 10.1002/gcc.10139

[42] Tosić N, Stojiljković M, Colović N, Colović M, Pavlović S. Acute myeloid leukemia with NUP98-HOXC13 fusion and FLT3 internal tandem duplication mutation: case report and literature review. Cancer genetics and cytogenetics. 2009;193(2):98–103. 19665070 10.1016/j.cancergencyto.2009.03.007

[43] Taketani T, Taki T, Shibuya N, Ito E, Kitazawa J, Terui K, Hayashi Y. The HOXD11 Gene Is Fused to the NUP98 Gene in Acute Myeloid Leukemia with t(2;11)(q31;p15)1. Cancer Research. 2002;62(1):33–7. 11782354

[44] Raza-Egilmez SZ, Jani-Sait SN, Grossi M, Higgins MJ, Shows TB, Aplan PD. NUP98-HOXD13 gene fusion in therapy-related acute myelogenous leukemia. Cancer Res. 1998;58(19):4269–73. 9766650

[45] Arai Y, Kyo T, Miwa H, Arai K, Kamada N, Kita K, Ohki M. Heterogeneous fusion transcripts involving the NUP98 gene and HOXD13 gene activation in a case of acute myeloid leukemia with the t(2;11)(q31;p15) translocation. Leukemia. 2000;14(9):1621–9. 10995009 10.1038/sj.leu.2401881

[46] Nakamura T, Yamazaki Y, Hatano Y, Miura I. NUP98 is fused to PMX1 homeobox gene in human acute myelogenous leukemia with chromosome translocation t(1;11)(q23;p15). Blood. 1999;94(2):741–7. 10397741

[47] Gervais C, Mauvieux L, Perrusson N, Hélias C, Struski S, Leymarie V, Lioure B, Lessard M. A new translocation t(9;11)(q34;p15) fuses NUP98 to a novel homeobox partner gene, PRRX2, in a therapy-related acute myeloid leukemia. Leukemia. 2005;19(1):145–8. 15496970 10.1038/sj.leu.2403565

[48] Jankovic D, Gorello P, Liu T, Ehret S, La Starza R, Desjobert C, Baty F, Brutsche M, Jayaraman P-S, Santoro A, Mecucci C, Schwaller J. Leukemogenic mechanisms and targets of a NUP98/HHEX fusion in acute myeloid leukemia. Blood. 2008;111(12):5672–82. 18388181 10.1182/blood-2007-09-108175

[49] Sorel N, Raimbault A, Brizard F, Depaire T, Pierini V, Dupraz C, Millot F, Mecucci C, Chomel JC. Identification and genetic characterization of a NUP98-HHEX molecular rearrangement in a pediatric acute myeloid leukemia. Leukemia & lymphoma. 2021;62(14):3531–5. 34399652 10.1080/10428194.2021.1966784

[50] Tao S, Song L, Deng Y, Chen Y, Shi Y, Gan Y, Deng Z, Ding B, He Z, Wang C, Yu L. Acute Myeloid Leukemia with NUP98-RARG Gene Fusion Similar to Acute Promyelocytic Leukemia: Case Report and Literature Review. OncoTargets and therapy. 2020;13:10559–66. 33116634 10.2147/OTT.S273172 PMC7574910

[51] Lisboa S, Cerveira N, Bizarro S, Correia C, Vieira J, Torres L, Mariz JM, Teixeira MR. POU1F1 is a novel fusion partner of NUP98 in acute myeloid leukemia with t(3;11)(p11;p15). Molecular Cancer. 2013;12(1):5. 23332017 10.1186/1476-4598-12-5 PMC3567982

[52] Ahuja HG, Hong J, Aplan PD, Tcheurekdjian L, Forman SJ, Slovak ML. t(9;11)(p22;p15) in acute myeloid leukemia results in a fusion between NUP98 and the gene encoding transcriptional coactivators p52 and p75-lens epithelium-derived growth factor (LEDGF). Cancer Res. 2000;60(22):6227–9. 11103774

[53] Hussey DJ, Moore S, Nicola M, Dobrovic A. Fusion of the NUP98 gene with the LEDGF/p52 gene defines a recurrent acute myeloid leukemia translocation. BMC Genetics. 2001;2(1):20. 11737860 10.1186/1471-2156-2-20 PMC60524

[54] Reader JC, Meekins JS, Gojo I, Ning Y. A novel NUP98-PHF23 fusion resulting from a cryptic translocation t(11;17)(p15;p13) in acute myeloid leukemia. Leukemia. 2007;21(4):842–4. 17287853 10.1038/sj.leu.2404579

[55] Togni M, Masetti R, Pigazzi M, Astolfi A, Zama D, Indio V, Serravalle S, Manara E, Bisio V, Rizzari C, Basso G, Pession A, Locatelli F. Identification of the NUP98-PHF23 fusion gene in pediatric cytogenetically normal acute myeloid leukemia by whole-transcriptome sequencing. Journal of Hematology & Oncology. 2015;8(1):69. 26066811 10.1186/s13045-015-0167-8 PMC4467064

[56] Cheng C-K, Chan H-Y, Yung Y-L, Wan TSK, Leung AWK, Li C-K, Tian K, Chan NPH, Cheung JS, Ng MHL. A novel NUP98-JADE2 fusion in a patient with acute myeloid leukemia resembling acute promyelocytic leukemia. Blood advances. 2022;6(2):410–5. 34673934 10.1182/bloodadvances.2021006064 PMC8791568

[57] de Rooij JDE, Hollink IHIM, Arentsen-Peters STCJM, van Galen JF, Berna Beverloo H, Baruchel A, Trka J, Reinhardt D, Sonneveld E, Zimmermann M, Alonzo TA, Pieters R, Meshinchi S, van den Heuvel-Eibrink MM, Zwaan CM. NUP98/JARID1A is a novel recurrent abnormality in pediatric acute megakaryoblastic leukemia with a distinct HOX gene expression pattern. Leukemia. 2013;27(12):2280–8. 23531517 10.1038/leu.2013.87

[58] Noort S, Wander P, Alonzo TA, Smith J, Ries RE, Gerbing RB, Dolman MEM, Locatelli F, Reinhardt D, Baruchel A, Stary J, Molenaar JJ, Stam RW, van den Heuvel-Eibrink MM, Zwaan MC, Meshinchi S. The clinical and biological characteristics of NUP98-KDM5A in pediatric acute myeloid leukemia. Haematologica. 2021;106(2):630–4. 32381579 10.3324/haematol.2019.236745 PMC7849578

[59] Jaju RJ, Fidler C, Haas OA, Strickson AJ, Watkins F, Clark K, Cross NCP, Cheng J-F, Aplan PD, Kearney L, Boultwood J, Wainscoat JS. A novel gene, NSD1, is fused to NUP98 in the t(5;11)(q35;p15.5) in de novo childhood acute myeloid leukemia. Blood. 2001;98(4):1264–7. 11493482 10.1182/blood.v98.4.1264

[60] Iacobucci I, Wen J, Meggendorfer M, Choi JK, Shi L, Pounds SB, Carmichael CL, Masih KE, Morris SM, Lindsley RC, Janke LJ, Alexander TB, Song G, Qu C, Li Y, Payne-Turner D, Tomizawa D, Kiyokawa N, Valentine M, Valentine V, Basso G, Locatelli F, Enemark EJ, Kham SKY, Yeoh AEJ, Ma X, Zhou X, Sioson E, Rusch M, Ries RE, Stieglitz E, Hunger SP, Wei AH, To LB, Lewis ID, D’Andrea RJ, Kile BT, Brown AL, Scott HS, Hahn CN, Marlton P, Pei D, Cheng C, Loh ML, Ebert BL, Meshinchi S, Haferlach T, Mullighan CG. Genomic subtyping and therapeutic targeting of acute erythroleukemia. Nature Genetics. 2019;51(4):694–704. 30926971 10.1038/s41588-019-0375-1 PMC6828160

[61] Wang T, Ni JB, Wang XY, Dai Y, Ma XL, Su YC, Gao YY, Chen X, Yuan LL, Liu HX. [Genetic characteristics and clinical outcomes of pediatric acute myeloid leukemia with NUP98-NSD1 fusion gene]. Zhonghua yi xue za zhi. 2019;99(36):2820–5. 31550809 10.3760/cma.j.issn.0376-2491.2019.36.005

[62] Rosati R, La Starza R, Veronese A, Aventin A, Schwienbacher C, Vallespi T, Negrini M, Martelli MF, Mecucci C. NUP98 is fused to the NSD3 gene in acute myeloid leukemia associated with t(8;11)(p11.2;p15). Blood. 2002;99(10):3857–60. 11986249 10.1182/blood.v99.10.3857

[63] Kaltenbach S, Soler G, Barin C, Gervais C, Bernard OA, Penard-Lacronique V, Romana SP. NUP98-MLL fusion in human acute myeloblastic leukemia. Blood. 2010;116(13):2332–5. 20558618 10.1182/blood-2010-04-277806

[64] Tosi S, Ballabio E, Teigler-Schlegel A, Boultwood J, Bruch J, Harbott J. Characterization of 6q abnormalities in childhood acute myeloid leukemia and identification of a novel t(6;11)(q24.1;p15.5) resulting in a NUP98-C6orf80 fusion in a case of acute megakaryoblastic leukemia. Genes Chromosomes Cancer. 2005;44(3):225–32. 16028218 10.1002/gcc.20233

[65] Petit A, Ragu C, Della-Valle V, Mozziconacci MJ, Lafage-Pochitaloff M, Soler G, Schluth C, Radford I, Ottolenghi C, Bernard OA, Penard-Lacronique V, Romana SP. NUP98–HMGB3: a novel oncogenic fusion. Leukemia. 2010;24(3):654–8. 19956199 10.1038/leu.2009.241

[66] Pan Q, Zhu Y-J, Gu B-W, Cai X, Bai X-T, Yuan H-Y, Zhu J, Chen Z, Xue Y-Q, Chen S-J. A New Fusion Gene NUP98-IQCG Identified in an Acute T/Myeloid Leukemia with t(3;11)(q29q13;p15) Translocation. Blood. 2007;110(11):1828. 10.1038/sj.onc.1210999 18084320

[67] Umeda M, Michmerhuizen N, Ma J, Westover T, Walsh MP, Song G, Mecucci C, Giacomo DD, Locatelli F, Masetti R, Bertuccio SN, Pigazzi M, Iacobucci I, Mullighan CG, Klco JM. AML-283 The Genetic Landscape of NUP98-Rearranged Pediatric Leukemia. Clinical Lymphoma Myeloma and Leukemia. 2022;22:S233.

[68] Bisio V, Pigazzi M, Manara E, Masetti R, Togni M, Astolfi A, Mecucci C, Zappavigna V, Salsi V, Merli P, Rizzari C, Fagioli F, Locatelli F, Basso G. NUP98 Fusion Proteins Are Recurrent Aberrancies in Childhood Acute Myeloid Leukemia: A Report from the AIEOP AML-2001–02 Study Group. Blood. 2014;124(21):1025-.

[69] Arai Y, Hosoda F, Kobayashi H, Arai K, Hayashi Y, Kamada N, Kaneko Y, Ohki M. The inv(11)(p15q22) chromosome translocation of de novo and therapy-related myeloid malignancies results in fusion of the nucleoporin gene, NUP98, with the putative RNA helicase gene, DDX10. Blood. 1997;89(11):3936–44. 9166830

[70] Hollink IHIM, van den Heuvel-Eibrink MM, Arentsen-Peters STCJM, Pratcorona M, Abbas S, Kuipers JE, van Galen JF, Beverloo HB, Sonneveld E, Kaspers G-JJL, Trka J, Baruchel A, Zimmermann M, Creutzig U, Reinhardt D, Pieters R, Valk PJM, Zwaan CM. NUP98/NSD1 characterizes a novel poor prognostic group in acute myeloid leukemia with a distinct HOX gene expression pattern. Blood. 2011;118(13):3645–56. 21813447 10.1182/blood-2011-04-346643

[71] Chen S, Xue Y, Chen Z, Guo Y, Wu Y, Pan J. Generation of the NUP98-TOP1 fusion transcript by the t(11;20) (p15;q11) in a case of acute monocytic leukemia. Cancer genetics and cytogenetics. 2003;140(2):153–6. 12645654 10.1016/s0165-4608(02)00642-8

[72] Nebral K, Schmidt HH, Haas OA, Strehl S. NUP98 Is Fused to Topoisomerase (DNA) IIβ 180 kDa (TOP2B) in a Patient with Acute Myeloid Leukemia with a New t(3;11)(p24;p15). Clinical Cancer Research. 2005;11(18):6489–94. 16166424 10.1158/1078-0432.CCR-05-0150

[73] Ishikawa M, Yagasaki F, Okamura D, Maeda T, Sugahara Y, Jinnai I, Bessho M. A novel gene, ANKRD28 on 3p25, is fused with NUP98 on 11p15 in a cryptic 3-way translocation of t(3;5;11)(p25;q35;p15) in an adult patient with myelodysplastic syndrome/acute myelogenous leukemia. Int J Hematol. 2007;86(3):238–45. 17988990 10.1532/IJH97.07054

[74] Gorello P, Brandimarte L, La Starza R, Pierini V, Bury L, Rosati R, Martelli MF, Vandenberghe P, Wlodarska I, Mecucci C. t(3;11)(q12;p15)/NUP98-LOC348801 fusion transcript in acute myeloid leukemia. Haematologica. 2008;93(9):1398–401. 18603550 10.3324/haematol.12945

[75] Liquori A, Ibañez M, Sargas C, Sanz MÁ, Barragán E, Cervera J. Acute Promyelocytic Leukemia: A Constellation of Molecular Events around a Single PML-RARA Fusion Gene. 2020;12(3):624. 10.3390/cancers12030624 PMC7139833 32182684

[76] Gurnari C, Voso MT, Girardi K, Mastronuzzi A, Strocchio L. Acute Promyelocytic Leukemia in Children: A Model of Precision Medicine and Chemotherapy-Free Therapy. International journal of molecular sciences. 2021;22(2). 10.3390/ijms22020642 PMC7826974 33440683

[77] de Rooij JDE, Masetti R, van den Heuvel-Eibrink MM, Cayuela J-M, Trka J, Reinhardt D, Rasche M, Sonneveld E, Alonzo TA, Fornerod M, Zimmermann M, Pigazzi M, Pieters R, Meshinchi S, Zwaan CM, Locatelli F. Recurrent abnormalities can be used for risk group stratification in pediatric AMKL: a retrospective intergroup study. Blood. 2016;127(26):3424–30. 27114462 10.1182/blood-2016-01-695551 PMC5161011

[78] Marceau-Renaut A, Duployez N, Ducourneau B, Labopin M, Petit A, Rousseau A, Geffroy S, Bucci M, Cuccuini W, Fenneteau O, Ruminy P, Nelken B, Ducassou S, Gandemer V, Leblanc T, Michel G, Bertrand Y, Baruchel A, Leverger G, Preudhomme C, Lapillonne H. Molecular Profiling Defines Distinct Prognostic Subgroups in Childhood AML: A Report From the French ELAM02 Study Group. HemaSphere. 2018;2(1):e31. 31723759 10.1097/HS9.0000000000000031 PMC6745946

[79] McNeer NA, Philip J, Geiger H, Ries RE, Lavallée V-P, Walsh M, Shah M, Arora K, Emde A-K, Robine N, Alonzo TA, Kolb EA, Gamis AS, Smith M, Gerhard DS, Guidry-Auvil J, Meshinchi S, Kentsis A. Genetic mechanisms of primary chemotherapy resistance in pediatric acute myeloid leukemia. Leukemia. 2019;33(8):1934–43. 30760869 10.1038/s41375-019-0402-3 PMC6687545

[80] Shimada A, Iijima-Yamashita Y, Tawa A, Tomizawa D, Yamada M, Norio S, Watanabe T, Taga T, Iwamoto S, Terui K, Moritake H, Kinoshita A, Takahashi H, Nakayama H, Koh K, Goto H, Kosaka Y, Saito AM, Kiyokawa N, Horibe K, Hara Y, Oki K, Hayashi Y, Tanaka S, Adachi S. Risk-stratified therapy for children with FLT3-ITD-positive acute myeloid leukemia: results from the JPLSG AML-05 study. International Journal of Hematology. 2018;107(5):586–95. 29330746 10.1007/s12185-017-2395-x

[81] Shiba N, Ichikawa H, Taki T, Park MJ, Jo A, Mitani S, Kobayashi T, Shimada A, Sotomatsu M, Arakawa H, Adachi S, Tawa A, Horibe K, Tsuchida M, Hanada R, Tsukimoto I, Hayashi Y. NUP98-NSD1 gene fusion and its related gene expression signature are strongly associated with a poor prognosis in pediatric acute myeloid leukemia. Genes Chromosomes Cancer. 2013;52(7):683–93. 23630019 10.1002/gcc.22064

[82] Xie W, Raess PW, Dunlap J, Hoyos CM, Li H, Li P, Swords R, Olson SB, Yang F, Anekpuritanang T, Hu S, Wiszniewska J, Fan G, Press RD, Moore SR. Adult acute myeloid leukemia patients with NUP98 rearrangement have frequent cryptic translocations and unfavorable outcome. Leukemia & lymphoma. 2022;63(8):1907–16. 35258401 10.1080/10428194.2022.2047672

[83] Niktoreh N, Walter C, Zimmermann M, von Neuhoff C, von Neuhoff N, Rasche M, Waack K, Creutzig U, Hanenberg H, Reinhardt D. Mutated WT1, FLT3-ITD, and NUP98-NSD1 Fusion in Various Combinations Define a Poor Prognostic Group in Pediatric Acute Myeloid Leukemia. Journal of oncology. 2019;2019:1609128. 31467532 10.1155/2019/1609128 PMC6699323

[84] Ostronoff F, Othus M, Gerbing RB, Loken MR, Raimondi SC, Hirsch BA, Lange BJ, Petersdorf S, Radich J, Appelbaum FR, Gamis AS, Alonzo TA, Meshinchi S. NUP98/NSD1 and FLT3/ITD coexpression is more prevalent in younger AML patients and leads to induction failure: a COG and SWOG report. Blood. 2014;124(15):2400–7. 25145343 10.1182/blood-2014-04-570929 PMC4192751

[85] Franks TM, Hetzer MW. The role of Nup98 in transcription regulation in healthy and diseased cells. Trends in cell biology. 2013;23(3):112–7. 23246429 10.1016/j.tcb.2012.10.013 PMC3622213

[86] Wang GG, Cai L, Pasillas MP, Kamps MP. NUP98–NSD1 links H3K36 methylation to Hox-A gene activation and leukaemogenesis. Nature Cell Biology. 2007;9(7):804–12. 17589499 10.1038/ncb1608

[87] Yung E, Sekulovic S, Argiropoulos B, Lai CK, Leung M, Berg T, Vollett S, Chang VC, Wan A, Wong S, Humphries RK. Delineating domains and functions of NUP98 contributing to the leukemogenic activity of NUP98-HOX fusions. Leuk Res. 2011;35(4):545–50. 21130494 10.1016/j.leukres.2010.10.006 PMC4072657

[88] Wang GG, Cai L, Pasillas MP, Kamps MP. NUP98-NSD1 links H3K36 methylation to Hox-A gene activation and leukaemogenesis. Nat Cell Biol. 2007;9(7):804–12. 17589499 10.1038/ncb1608

[89] Gurevich RM, Aplan PD, Humphries RK. NUP98-topoisomerase I acute myeloid leukemia-associated fusion gene has potent leukemogenic activities independent of an engineered catalytic site mutation. Blood. 2004;104(4):1127–36. 15100157 10.1182/blood-2003-10-3550

[90] Hirose K, Abramovich C, Argiropoulos B, Humphries RK. Leukemogenic properties of NUP98-PMX1 are linked to NUP98 and homeodomain sequence functions but not to binding properties of PMX1 to serum response factor. Oncogene. 2008;27(46):6056–67. 18604245 10.1038/onc.2008.210

[91] Pan M, Zhang Q, Liu P, Huang J, Wang Y, Chen S. Inhibition of the nuclear export of p65 and IQCG in leukemogenesis by NUP98-IQCG. Frontiers of Medicine. 2016;10(4):410–9. 27864780 10.1007/s11684-016-0489-0

[92] Takeda A, Sarma NJ, Abdul-Nabi AM, Yaseen NR. Inhibition of CRM1-mediated nuclear export of transcription factors by leukemogenic NUP98 fusion proteins. The Journal of biological chemistry. 2010;285(21):16248–57. 20233715 10.1074/jbc.M109.048785 PMC2871492

[93] Pan MM, Zhang QY, Wang YY, Liu P, Ren RB, Huang JY, Chen LT, Xi XD, Chen Z, Chen SJ. Human NUP98-IQCG fusion protein induces acute myelomonocytic leukemia in mice by dysregulating the Hox/Pbx3 pathway. Leukemia. 2016;30(7):1590–3. 26675333 10.1038/leu.2015.347

[94] Boija A, Klein IA, Sabari BR, Dall’Agnese A, Coffey EL, Zamudio AV, Li CH, Shrinivas K, Manteiga JC, Hannett NM, Abraham BJ, Afeyan LK, Guo YE, Rimel JK, Fant CB, Schuijers J, Lee TI, Taatjes DJ, Young RA. Transcription Factors Activate Genes through the Phase-Separation Capacity of Their Activation Domains. Cell. 2018;175(7):1842–55.e16. 30449618 10.1016/j.cell.2018.10.042 PMC6295254

[95] Ahn JH, Davis ES, Daugird TA, Zhao S, Quiroga IY, Uryu H, Li J, Storey AJ, Tsai Y-H, Keeley DP, Mackintosh SG, Edmondson RD, Byrum SD, Cai L, Tackett AJ, Zheng D, Legant WR, Phanstiel DH, Wang GG. Phase separation drives aberrant chromatin looping and cancer development. Nature. 2021;595(7868):591–5. 34163069 10.1038/s41586-021-03662-5 PMC8647409

[96] Chandra B, Michmerhuizen NL, Shirnekhi HK, Tripathi S, Pioso BJ, Baggett DW, Mitrea DM, Iacobucci I, White MR, Chen J, Park CG, Wu H, Pounds S, Medyukhina A, Khairy K, Gao Q, Qu C, Abdelhamed S, Gorman SD, Bawa S, Maslanka C, Kinger S, Dogra P, Ferrolino MC, Di Giacomo D, Mecucci C, Klco JM, Mullighan CG, Kriwacki RW. Phase Separation Mediates NUP98 Fusion Oncoprotein Leukemic Transformation. Cancer Discov. 2022;12(4):1152–69. 34903620 10.1158/2159-8290.CD-21-0674 PMC8983581

[97] Terlecki-Zaniewicz S, Humer T, Eder T, Schmoellerl J, Heyes E, Manhart G, Kuchynka N, Parapatics K, Liberante FG, Müller AC, Tomazou EM, Grebien F. Biomolecular condensation of NUP98 fusion proteins drives leukemogenic gene expression. Nature structural & molecular biology. 2021;28(2):190–201. 10.1038/s41594-020-00550-w PMC7116736 33479542

[98] Lawrence HJ, Christensen J, Fong S, Hu YL, Weissman I, Sauvageau G, Humphries RK, Largman C. Loss of expression of the Hoxa-9 homeobox gene impairs the proliferation and repopulating ability of hematopoietic stem cells. Blood. 2005;106(12):3988–94. 16091451 10.1182/blood-2005-05-2003 PMC1895111

[99] Pineault N, Helgason CD, Lawrence HJ, Humphries RK. Differential expression of Hox, Meis1, and Pbx1 genes in primitive cells throughout murine hematopoietic ontogeny. Exp Hematol. 2002;30(1):49–57. 11823037 10.1016/s0301-472x(01)00757-3

[100] Kroon E, Krosl J, Thorsteinsdottir U, Baban S, Buchberg AM, Sauvageau G. Hoxa9 transforms primary bone marrow cells through specific collaboration with Meis1a but not Pbx1b. Embo j. 1998;17(13):3714–25. 9649441 10.1093/emboj/17.13.3714 PMC1170707

[101] Zhang T, Cooper S, Brockdorff N. The interplay of histone modifications - writers that read. EMBO reports. 2015;16(11):1467–81. 26474904 10.15252/embr.201540945 PMC4641500

[102] Wang GG, Song J, Wang Z, Dormann HL, Casadio F, Li H, Luo JL, Patel DJ, Allis CD. Haematopoietic malignancies caused by dysregulation of a chromatin-binding PHD finger. Nature. 2009;459(7248):847–51. 19430464 10.1038/nature08036 PMC2697266

[103] Kuo AJ, Cheung P, Chen K, Zee BM, Kioi M, Lauring J, Xi Y, Park BH, Shi X, Garcia BA, Li W, Gozani O. NSD2 links dimethylation of histone H3 at lysine 36 to oncogenic programming. Molecular cell. 2011;44(4):609–20. 22099308 10.1016/j.molcel.2011.08.042 PMC3222870

[104] Gough SM, Lee F, Yang F, Walker RL, Zhu YJ, Pineda M, Onozawa M, Chung YJ, Bilke S, Wagner EK, Denu JM, Ning Y, Xu B, Wang GG, Meltzer PS, Aplan PD. NUP98-PHF23 is a chromatin-modifying oncoprotein that causes a wide array of leukemias sensitive to inhibition of PHD histone reader function. Cancer Discov. 2014;4(5):564–77. 24535671 10.1158/2159-8290.CD-13-0419 PMC4018760

[105] Zhang Y, Guo Y, Gough SM, Zhang J, Vann KR, Li K, Cai L, Shi X, Aplan PD, Wang GG, Kutateladze TG. Mechanistic insights into chromatin targeting by leukemic NUP98-PHF23 fusion. Nature Communications. 2020;11(1):3339. 10.1038/s41467-020-17098-4 PMC7335091 32620764

[106] Kroon E, Thorsteinsdottir U, Mayotte N, Nakamura T, Sauvageau G. NUP98-HOXA9 expression in hemopoietic stem cells induces chronic and acute myeloid leukemias in mice. Embo j. 2001;20(3):350–61. 11157742 10.1093/emboj/20.3.350 PMC133485

[107] Pineault N, Abramovich C, Humphries RK. Transplantable cell lines generated with NUP98-Hox fusion genes undergo leukemic progression by Meis1 independent of its binding to DNA. Leukemia. 2005;19(4):636–43. 15744344 10.1038/sj.leu.2403696

[108] Pineault N, Buske C, Feuring-Buske M, Abramovich C, Rosten P, Hogge DE, Aplan PD, Humphries RK. Induction of acute myeloid leukemia in mice by the human leukemia-specific fusion gene NUP98-HOXD13 in concert with Meis1. Blood. 2003;101(11):4529–38. 12543865 10.1182/blood-2002-08-2484

[109] Calvo KR, Sykes DB, Pasillas MP, Kamps MP. Nup98-HoxA9 immortalizes myeloid progenitors, enforces expression of Hoxa9, Hoxa7 and Meis1, and alters cytokine-specific responses in a manner similar to that induced by retroviral co-expression of Hoxa9 and Meis1. Oncogene. 2002;21(27):4247–56. 12082612 10.1038/sj.onc.1205516

[110] Salsi V, Ferrari S, Gorello P, Fantini S, Chiavolelli F, Mecucci C, Zappavigna V. NUP98 Fusion Oncoproteins Promote Aneuploidy by Attenuating the Mitotic Spindle Checkpoint. Cancer Research. 2014;74(4):1079–90. 24371226 10.1158/0008-5472.CAN-13-0912

[111] Ricke RM, van Ree JH, van Deursen JM. Whole chromosome instability and cancer: a complex relationship. Trends in genetics : TIG. 2008;24(9):457–66. 18675487 10.1016/j.tig.2008.07.002 PMC2594012

[112] Bharadwaj R, Yu H. The spindle checkpoint, aneuploidy, and cancer. Oncogene. 2004;23(11):2016–27. 15021889 10.1038/sj.onc.1207374

[113] Salsi V, Fantini S, Zappavigna V. NUP98 fusion oncoproteins interact with the APC/C(Cdc20) as a pseudosubstrate and prevent mitotic checkpoint complex binding. Cell cycle (Georgetown, Tex). 2016;15(17):2275–87. 27097363 10.1080/15384101.2016.1172156 PMC5004700

[114] Taketani T, Taki T, Nakamura T, Kobayashi Y, Ito E, Fukuda S, Yamaguchi S, Hayashi Y. High frequencies of simultaneous FLT3-ITD, WT1 and KIT mutations in hematological malignancies with NUP98-fusion genes. Leukemia. 2010;24(11):1975–7. 20861915 10.1038/leu.2010.207

[115] Guan W, Zhou L, Li Y, Yang E, Liu Y, Lv N, Fu L, Ding Y, Wang N, Fang N, Liu Q, Wang B, Li F, Zhang J, Wang M, Wang L, Jing Y, Li Y, Yu L. Profiling of somatic mutations and fusion genes in acute myeloid leukemia patients with FLT3-ITD or FLT3-TKD mutation at diagnosis reveals distinct evolutionary patterns. Exp Hematol Oncol. 2021;10(1):27. 33836835 10.1186/s40164-021-00207-4 PMC8033687

[116] Fang Y, Han X, Shen L, Hou J. NUP98-HOXA9 Bearing Acute Myeloid Leukemia. Blood. 2022;140(Supplement 1):11614-.

[117] Chou WC, Chen CY, Hou HA, Lin LI, Tang JL, Yao M, Tsay W, Ko BS, Wu SJ, Huang SY, Hsu SC, Chen YC, Huang YN, Tseng MH, Huang CF, Tien HF. Acute myeloid leukemia bearing t(7;11)(p15;p15) is a distinct cytogenetic entity with poor outcome and a distinct mutation profile: comparative analysis of 493 adult patients. Leukemia. 2009;23(7):1303–10. 19225539 10.1038/leu.2009.25

[118] Lavallée VP, Lemieux S, Boucher G, Gendron P, Boivin I, Girard S, Hébert J, Sauvageau G. Identification of MYC mutations in acute myeloid leukemias with NUP98–NSD1 translocations. Leukemia. 2016;30(7):1621–4. 26859078 10.1038/leu.2016.19

[119] Cui J, Xie J, Qin L, Chen S, Zhao Y, Wu D. A unique acute myeloid leukemia patient with cryptic NUP98-NSD1 gene and ASXL1 mutation. Leukemia & lymphoma. 2016;57(1):196–8. 25860235 10.3109/10428194.2015.1037755

[120] Kelly LM, Gilliland DG. Genetics of myeloid leukemias. Annual review of genomics and human genetics. 2002;3:179–98. 10.1146/annurev.genom.3.032802.115046 12194988

[121] Takahashi S Current findings for recurring mutations in acute myeloid leukemia. J Hematol Oncol. 2011;4:36. 21917154 10.1186/1756-8722-4-36 PMC3180439

[122] Ahuja HG, Popplewell L, Tcheurekdjian L, Slovak ML. NUP98 gene rearrangements and the clonal evolution of chronic myelogenous leukemia. Genes Chromosomes Cancer. 2001;30(4):410–5. 11241795 10.1002/1098-2264(2001)9999:9999<::aid-gcc1108>3.0.co;2-9

[123] Yamamoto K, Nakamura Y, Nakamura Y, Saito K, Furusawa S. Expression of the NUP98/HOXA9 fusion transcript in the blast crisis of Philadelphia chromosome-positive chronic myelogenous leukaemia with t(7;11)(p15;p15). 2000;109(2):423–6. 10.1046/j.1365-2141.2000.02003.x 10848835

[124] Dash AB, Williams IR, Kutok JL, Tomasson MH, Anastasiadou E, Lindahl K, Li S, Van Etten RA, Borrow J, Housman D, Druker B, Gilliland DG. A murine model of CML blast crisis induced by cooperation between BCR/ABL and NUP98/HOXA9. Proc Natl Acad Sci U S A. 2002;99(11):7622–7. 12032333 10.1073/pnas.102583199 PMC124303

[125] Yamamoto M, Kakihana K, Kurosu T, Murakami N, Miura O. Clonal evolution with inv(11)(p15q22) and NUP98/DDX10 fusion gene in imatinib-resistant chronic myelogenous leukemia. Cancer genetics and cytogenetics. 2005;157(2):104–8. 15721630 10.1016/j.cancergencyto.2004.06.014

[126] Mohanty S, Heuser M. Mouse Models of Frequently Mutated Genes in Acute Myeloid Leukemia. Cancers (Basel). 2021;13(24). 10.3390/cancers13246192 PMC8699817 34944812

[127] Greenblatt S, Li L, Slape C, Nguyen B, Novak R, Duffield A, Huso D, Desiderio S, Borowitz MJ, Aplan P, Small D. Knock-in of a FLT3/ITD mutation cooperates with a NUP98-HOXD13 fusion to generate acute myeloid leukemia in a mouse model. Blood. 2012;119(12):2883–94. 22323452 10.1182/blood-2011-10-382283 PMC3327463

[128] Mohanty S, Jyotsana N, Sharma A, Kloos A, Gabdoulline R, Othman B, Lai CK, Schottmann R, Mandhania M, Schmoellerl J, Grebien F, Ramsay E, Thomas A, Vornlocher H-P, Ganser A, Thol F, Heuser M. Targeted Inhibition of the NUP98-NSD1 Fusion Oncogene in Acute Myeloid Leukemia. 2020;12(10):2766. 10.3390/cancers12102766 PMC7600396 32993115

[129] Thanasopoulou A, Tzankov A, Schwaller J. Potent co-operation between the NUP98-NSD1 fusion and the FLT3-ITD mutation in acute myeloid leukemia induction. Haematologica. 2014;99(9):1465–71. 24951466 10.3324/haematol.2013.100917 PMC4562535

[130] Matsukawa T, Yin M, Nigam N, Negi V, Li L, Small D, Zhu YJ, Walker RL, Meltzer PS, Aplan PD. NUP98::Nsd1 and FLT3-ITD collaborate to generate acute myeloid leukemia. Leukemia. 2023. 10.1038/s41375-023-01913-0 PMC10317830 37147424

[131] Nakamura T Retroviral insertional mutagenesis identifies oncogene cooperation. 2005;96(1):7–12. 10.1111/j.1349-7006.2005.00011.x PMC11158914 15649248

[132] Slape C, Hartung H, Lin YW, Bies J, Wolff L, Aplan PD. Retroviral insertional mutagenesis identifies genes that collaborate with NUP98-HOXD13 during leukemic transformation. Cancer Res. 2007;67(11):5148–55. 17545593 10.1158/0008-5472.CAN-07-0075 PMC1950322

[133] Slape C, Liu LY, Beachy S, Aplan PD. Leukemic transformation in mice expressing a NUP98-HOXD13 transgene is accompanied by spontaneous mutations in Nras, Kras, and Cbl. Blood. 2008;112(5):2017–9. 18566322 10.1182/blood-2008-01-135186 PMC2518902

[134] Stahl M, Tallman MS. Acute promyelocytic leukemia (APL): remaining challenges towards a cure for all. Leukemia & lymphoma. 2019;60(13):3107–15. 31842650 10.1080/10428194.2019.1613540 PMC7479633

[135] Pikman Y, Stegmaier K. Targeted therapy for fusion-driven high-risk acute leukemia. Blood. 2018;132(12):1241–7. 30049809 10.1182/blood-2018-04-784157 PMC6148448

[136] Jyotsana N, Sharma A, Chaturvedi A, Budida R, Scherr M, Kuchenbauer F, Lindner R, Noyan F, Sühs KW, Stangel M, Grote-Koska D, Brand K, Vornlocher HP, Eder M, Thol F, Ganser A, Humphries RK, Ramsay E, Cullis P, Heuser M. Lipid nanoparticle-mediated siRNA delivery for safe targeting of human CML in vivo. Annals of hematology. 2019;98(8):1905–18. 31104089 10.1007/s00277-019-03713-y PMC7116733

[137] Jyotsana N, Sharma A, Chaturvedi A, Scherr M, Kuchenbauer F, Sajti L, Barchanski A, Lindner R, Noyan F, Sühs KW, Grote-Koska D, Brand K, Vornlocher HP, Stanulla M, Bornhauser B, Bourquin JP, Eder M, Thol F, Ganser A, Humphries RK, Ramsay E, Cullis P, Heuser M. RNA interference efficiently targets human leukemia driven by a fusion oncogene in vivo. Leukemia. 2018;32(1):224–6. 28827563 10.1038/leu.2017.269 PMC5702262

[138] Issa H, Swart LE, Rasouli M, Ashtiani M, Nakjang S, Jyotsana N, Schuschel K, Heuser M, Blair H, Heidenreich O. Nanoparticle-mediated targeting of the fusion gene RUNX1/ETO in t(8;21)-positive acute myeloid leukaemia. Leukemia. 2023;37(4):820–34. 36823395 10.1038/s41375-023-01854-8 PMC10079536

[139] Schmoellerl J, Barbosa IAM, Eder T, Brandstoetter T, Schmidt L, Maurer B, Troester S, Pham HTT, Sagarajit M, Ebner J, Manhart G, Aslan E, Terlecki-Zaniewicz S, Van der Veen C, Hoermann G, Duployez N, Petit A, Lapillonne H, Puissant A, Itzykson R, Moriggl R, Heuser M, Meisel R, Valent P, Sexl V, Zuber J, Grebien F. CDK6 is an essential direct target of NUP98 fusion proteins in acute myeloid leukemia. Blood. 2020;136(4):387–400. 32344427 10.1182/blood.2019003267 PMC7115844

[140] Zhang M, Zhang L, Hei R, Li X, Cai H, Wu X, Zheng Q, Cai C. CDK inhibitors in cancer therapy, an overview of recent development. American journal of cancer research. 2021;11(5):1913–35. 34094661 PMC8167670

[141] Krivtsov AV, Evans K, Gadrey JY, Eschle BK, Hatton C, Uckelmann HJ, Ross KN, Perner F, Olsen SN, Pritchard T, McDermott L, Jones CD, Jing D, Braytee A, Chacon D, Earley E, McKeever BM, Claremon D, Gifford AJ, Lee HJ, Teicher BA, Pimanda JE, Beck D, Perry JA, Smith MA, McGeehan GM, Lock RB, Armstrong SA. A Menin-MLL Inhibitor Induces Specific Chromatin Changes and Eradicates Disease in Models of MLL-Rearranged Leukemia. Cancer Cell. 2019;36(6):660–73.e11. 31821784 10.1016/j.ccell.2019.11.001 PMC7227117

[142] Uckelmann HJ, Kim SM, Wong EM, Hatton C, Giovinazzo H, Gadrey JY, Krivtsov AV, Rücker FG, Döhner K, McGeehan GM, Levine RL, Bullinger L, Vassiliou GS, Armstrong SA. Therapeutic targeting of preleukemia cells in a mouse model of NPM1 mutant acute myeloid leukemia. Science. 2020;367(6477):586–90. 32001657 10.1126/science.aax5863 PMC7754791

[143] Pommert L, Tarlock K. The evolution of targeted therapy in pediatric AML: gemtuzumab ozogamicin, FLT3/IDH/BCL2 inhibitors, and other therapies. Hematology American Society of Hematology Education Program. 2022;2022(1):603–10. 36485125 10.1182/hematology.2022000358 PMC9819987

[144] Heikamp EB, Henrich JA, Perner F, Wong EM, Hatton C, Wen Y, Barwe SP, Gopalakrishnapillai A, Xu H, Uckelmann HJ, Takao S, Kazansky Y, Pikman Y, McGeehan GM, Kolb EA, Kentsis A, Armstrong SA. The menin-MLL1 interaction is a molecular dependency in NUP98-rearranged AML. Blood. 2022;139(6):894–906. 34582559 10.1182/blood.2021012806 PMC8832476

[145] Issa GC, Ravandi F, DiNardo CD, Jabbour E, Kantarjian HM, Andreeff M. Therapeutic implications of menin inhibition in acute leukemias. Leukemia. 2021;35(9):2482–95. 34131281 10.1038/s41375-021-01309-y

[146] Issa GC, Aldoss I, DiPersio J, Cuglievan B, Stone R, Arellano M, Thirman MJ, Patel MR, Dickens DS, Shenoy S, Shukla N, Kantarjian H, Armstrong SA, Perner F, Perry JA, Rosen G, Bagley RG, Meyers ML, Ordentlich P, Gu Y, Kumar V, Smith S, McGeehan GM, Stein EM. The menin inhibitor revumenib in KMT2A-rearranged or NPM1-mutant leukaemia. Nature. 2023;615(7954):920–4. 36922593 10.1038/s41586-023-05812-3 PMC10060155

[147] Perner F, Stein EM, Wenge DV, Singh S, Kim J, Apazidis A, Rahnamoun H, Anand D, Marinaccio C, Hatton C, Wen Y, Stone RM, Schaller D, Mowla S, Xiao W, Gamlen HA, Stonestrom AJ, Persaud S, Ener E, Cutler JA, Doench JG, McGeehan GM, Volkamer A, Chodera JD, Nowak RP, Fischer ES, Levine RL, Armstrong SA, Cai SF. MEN1 mutations mediate clinical resistance to menin inhibition. Nature. 2023;615(7954):913–9. 36922589 10.1038/s41586-023-05755-9 PMC10157896

[148] Michmerhuizen NL, Umeda M, Iacobucci I, Arthur B, Di Giacomo D, Hiltenbrand R, Portola P, Abdelhamed S, Huang H, Zhou P, Long L, Shi H, Sun Y, Papachristou EK, Chilamakuri C, D’Santos CS, Chi H, Klco JM, Mullighan CG. Histone Acetyltransferases Are Critical Interacting Proteins in NUP98-Rearranged Acute Myeloid Leukemia. Blood. 2022;140(Supplement 1):2970–1.

[149] Oka M, Mura S, Yamada K, Sangel P, Hirata S, Maehara K, Kawakami K, Tachibana T, Ohkawa Y, Kimura H, Yoneda Y. Chromatin-prebound Crm1 recruits Nup98-HoxA9 fusion to induce aberrant expression of Hox cluster genes. eLife. 2016;5:e09540. 26740045 10.7554/eLife.09540 PMC4718815

[150] Ren Z, Kim A, Huang YT, Pi WC, Gong W, Yu X, Qi J, Jin J, Cai L, Roeder RG, Chen WY, Wang GG. A PRC2-Kdm5b axis sustains tumorigenicity of acute myeloid leukemia. Proc Natl Acad Sci U S A. 2022;119(9). 10.1073/pnas.2122940119 PMC8892512 35217626

[151] Gan L, Yang Y, Li Q, Feng Y, Liu T, Guo W. Epigenetic regulation of cancer progression by EZH2: from biological insights to therapeutic potential. Biomarker Research. 2018;6(1):10. 29556394 10.1186/s40364-018-0122-2 PMC5845366

[152] Straining R, Eighmy W. Tazemetostat: EZH2 Inhibitor. Journal of the advanced practitioner in oncology. 2022;13(2):158–63. 35369397 10.6004/jadpro.2022.13.2.7 PMC8955562

[153] Kivioja JL, Thanasopoulou A, Kumar A, Kontro M, Yadav B, Majumder MM, Javarappa KK, Eldfors S, Schwaller J, Porkka K, Heckman CA. Dasatinib and navitoclax act synergistically to target NUP98-NSD1(+)/FLT3-ITD(+) acute myeloid leukemia. Leukemia. 2019;33(6):1360–72. 30568173 10.1038/s41375-018-0327-2

[154] Harada K, Doki N, Aoki J, Mori J, Machida S, Masuko M, Uchida N, Najima Y, Fukuda T, Kanamori H, Ogawa H, Ota S, Ogawa K, Takahashi S, Kasai M, Maeda A, Nagafuji K, Kawakita T, Ichinohe T, Atsuta Y. Outcomes after allogeneic hematopoietic stem cell transplantation in patients with acute myeloid leukemia harboring t(7;11)(p15;p15). Haematologica. 2018;103(2):e69–e72. 29146707 10.3324/haematol.2017.179804 PMC5792289

[155] Békés M, Langley DR, Crews CM. PROTAC targeted protein degraders: the past is prologue. Nature Reviews Drug Discovery. 2022;21(3):181–200. 35042991 10.1038/s41573-021-00371-6 PMC8765495

[156] Wang Y, Shi T, Song X, Liu B, Wei J. Gene fusion neoantigens: Emerging targets for cancer immunotherapy. Cancer letters. 2021;506:45–54. 33675984 10.1016/j.canlet.2021.02.023

[157] Yang W, Lee KW, Srivastava RM, Kuo F, Krishna C, Chowell D, Makarov V, Hoen D, Dalin MG, Wexler L, Ghossein R, Katabi N, Nadeem Z, Cohen MA, Tian SK, Robine N, Arora K, Geiger H, Agius P, Bouvier N, Huberman K, Vanness K, Havel JJ, Sims JS, Samstein RM, Mandal R, Tepe J, Ganly I, Ho AL, Riaz N, Wong RJ, Shukla N, Chan TA, Morris LGT. Immunogenic neoantigens derived from gene fusions stimulate T cell responses. Nat Med. 2019;25(5):767–75. 31011208 10.1038/s41591-019-0434-2 PMC6558662

[158] Okamoto K, Imamura T, Tanaka S, Urata T, Yoshida H, Shiba N, Iehara T. The Nup98::Nsd1 fusion gene induces CD123 expression in 32D cells. Int J Hematol. 2023. 10.1007/s12185-023-03612-z 37173550

[159] Thiollier C, Lopez CK, Gerby B, Ignacimouttou C, Poglio S, Duffourd Y, Guégan J, Rivera-Munoz P, Bluteau O, Mabialah V, Diop M, Wen Q, Petit A, Bauchet AL, Reinhardt D, Bornhauser B, Gautheret D, Lecluse Y, Landman-Parker J, Radford I, Vainchenker W, Dastugue N, de Botton S, Dessen P, Bourquin JP, Crispino JD, Ballerini P, Bernard OA, Pflumio F, Mercher T. Characterization of novel genomic alterations and therapeutic approaches using acute megakaryoblastic leukemia xenograft models. J Exp Med. 2012;209(11):2017–31. 23045605 10.1084/jem.20121343 PMC3478932

[160] Mohanty S, Jyotsana N, Sharma A, Othman B, Kloos A, Mandhania M, Schottmann R, Ramsay E, Vornlocher H-P, Ganser A, Thol F, Heuser M. Targeted Inhibition of the NUP98-NSD1 Fusion Oncogene in AML. Blood. 2019;134:2545.

